# Enhancing FAIRdata by providing digital workflows from data generation to the publication of data: an open source approach described for cyclic voltammetry†

**DOI:** 10.1039/d4sc08620a

**Published:** 2025-02-05

**Authors:** David Herrmann, Patrick Hodapp, Martin Starman, Pei-Chi Huang, Chia-Lin Lin, Lan B. Q. Le, Tillmann G. Fischer, Claudia Bizzarri, Philipp Röse, Niklas Oppel, Jochen Klar, Pierre Tremouilhac, Laura Holzhauer, Sonja Herres-Pawlis, Alexander Hoffmann, Tobias Seitz, Alrik Dorn, Kirsten Zeitler, Nicole Jung, Stefan Bräse

**Affiliations:** a Institute of Biological and Chemical Systems, Functional Molecular Systems (IBCS), Karlsruhe Institute of Technology Kaiserstraße 12 76131 Karlsruhe Germany nicole.jung@kit.edu stefan.braese@kit.edu; b Institute for Biological Interfaces 3 – Soft Matter Laboratory (IBG 3 – SML), Karlsruhe Institute of Technology Kaiserstraße 12 76131 Karlsruhe Germany; c Independent Developer Cheruskerstr. 3 10829 Berlin Germany https://jochenklar.de; d Institute of Organic Chemistry (IOC), Karlsruhe Institute of Technology Kaiserstraße 12 76131 Karlsruhe Germany; e Institute for Applied Materials – Electrochemical Technologies (IAM-ET), Karlsruhe Institute of Technology Kaiserstraße 12, Adenauerring 20b 76131 Karlsruhe Germany; f Department Exposure Science, Helmholtz Centre for Environmental Research (UFZ) Permoserstraße 15 04318 Leipzig Germany; g RWTH Aachen University, Institute of Inorganic Chemistry Landoltweg 1a 52074 Aachen Germany; h Institute of Organic Chemistry, Leipzig University Johannisallee 29 04103 Leipzig Germany; i Karlsruhe Nano Micro Facility (KNMFi), Karlsruhe Institute of Technology Kaiserstraße 12 76131 Karlsruhe Germany

## Abstract

Analytical data in chemistry and other disciplines is usually generated in different formats and lacks common data and metadata standards that are necessary for a FAIR handling of research data. In the work presented herein, we describe a workflow that uses non-standardized, in some cases proprietary, data formats from cyclic voltammetry measurements coming from individual devices as an instructive example, to yield open, standardized data that are annotated with rich metadata. The presented workflow includes concepts, software and infrastructure that can be used to support the whole data life cycle from the measurement of data to the publication of data and metadata in repositories. Components used for this workflow were made available as open source, allowing the re-use of this approach in other laboratories. The methods described for cyclic voltammetry can be adapted and used for other measurements and experimental data collections, allowing for an easy way to integrate new methods for digitalized research and FAIR data management.

## Abstract 2

The generation of FAIR data in experimental sciences, *e.g.* for cyclic voltammetry datasets, involves multiple steps, including collecting data from devices, processing the data in an ELN environment, and publishing it in a repository.

## Graphical abstract

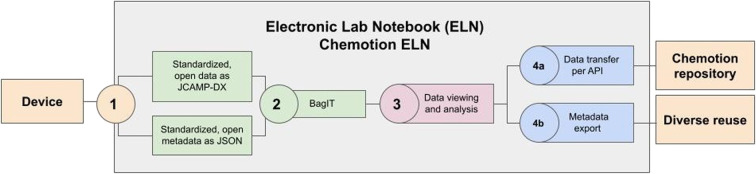

## Background

**Fig. 1:**
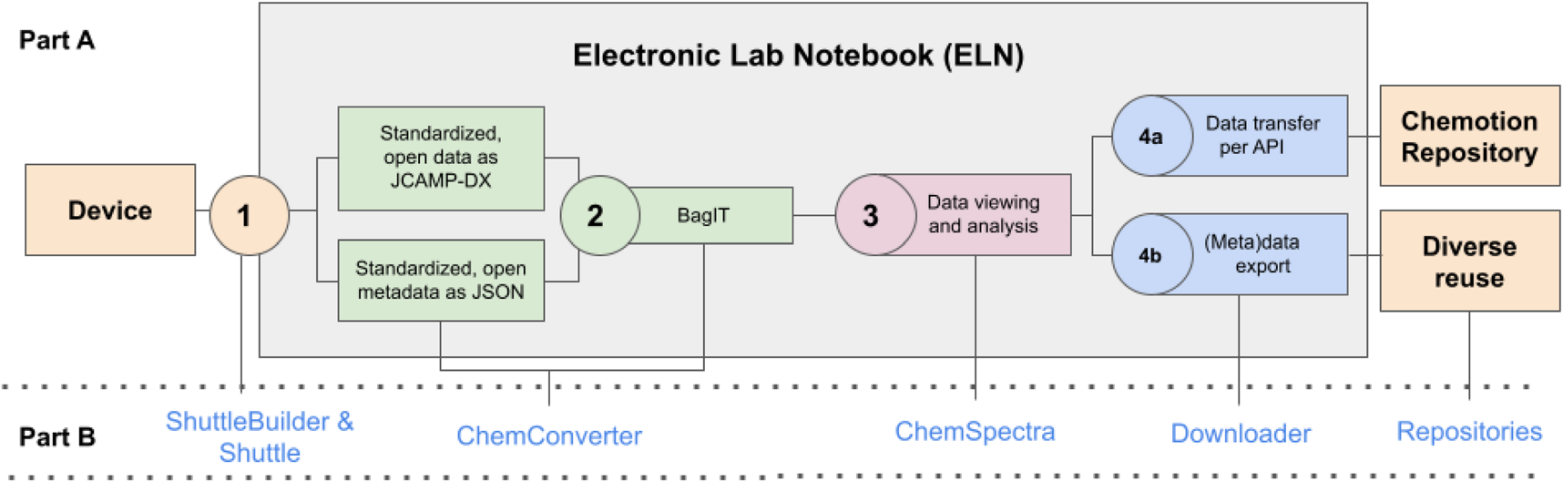
Schematic summary of the most important steps to gain FAIR data by collecting data from devices, processing them in an ELN and the final publication in a repository. The upper part (A) describes the single steps 1–4 that need to be implemented depending on the different workspaces device, ELN and repository. Part (B) gives the assignment to the tools that are used to enable the needed steps (described in the following sections and ESI, Section 1†).

The availability of FAIR data^1^ is of importance for scientists, but also for the community as a whole. Currently, most of the data in experimental sciences is not generated, stored or published in a FAIR manner. Therefore, concepts to generate FAIR data are needed for an efficient strategy that facilitates the work of scientists and reduces the current burden of work with data.^2,3^ This means, in consequence, that novel methods for the full digital handling of data have to be established.^4^ Only if data is available in a digital, readable form at the earliest possible point in time in the data life cycle, the concepts of FAIR data can be easily implemented. Steps towards a “FAIRer” generation and storage of the data provided by scientific instruments can be (a) the improvement of accessibility of the data, (b) the conversion of proprietary and non-standardized data to machine-readable and standardized data, (c) the annotation of data with metadata and (d) the provision of software to easily analyze the data and return the results in machine-readable and standardized formats. Dedicated LIMS^5^ (Laboratory and Information Management Systems) such as solutions from ThermoFisher,^6^ Benchling,^7^ Agilent^8^ and many others or ELNs (Electronic Lab Notebooks)^9^ such as eLabFTW,^10,11^ Labfolder,^11^ eLabJournal (partial commercial),^12^ and SciNote (partial commercial)^13^ can organize at least some aspects of the mentioned steps. Most of these systems can be used for different disciplines, and some of them also offer the option to systematically connect devices.^14^ Traditionally, for the chemical, biochemical and pharmaceutical industry, the need for functionality of a LIMS and ELN, *i.e.* the digital access to device information and the integration with the work documentation is very high. Many large companies in those areas invest in LIMS and ELN and benefit from a broad portfolio of tools to transfer and re-use data, while academic institutions and many small companies may lack the necessary finances to buy and maintain such systems. The implementation of open source solutions could be an option for those companies and academia with limited budgets, but systems that offer the broad spectrum of functionality, necessary for many use-cases, are still rare. Recently, open source solutions to the control of devices and the use of data produced by lab devices for AI-supported automation were presented.^15,16^ These examples can offer suitable options to enable the communication of devices with additional moderating infrastructure. Currently, these developments lack solutions to harmonize data and metadata within the scientific community if (meta)data are not accessible through self-built devices or standardized protocols such as SILA. Additionally, the embedding of the available workflows into the data lifecycle (including visualization, editing, documentation and analysis of data and metadata) was not gained yet, making the solutions hard to adapt for standard chemistry labs. Referring to systems offering such an environment that meets the requirements of research data management, only few open source systems offer device integration and documentation functions in chemistry and biology, for example Chemotion^17,18^ and OpenBIS.^19,20^ To the best of our knowledge, the available systems so far only offer incomprehensive solutions, lacking at least one step in the typical workflow (compare Fig. 1) that usually consists of (1) digital data acquisition, (2) data transfer from devices to an ELN (or any other digital work environment for documentation), (3) assignment of the data to the experiment/sample, (4) processing of the data to a readable and standardized file format, (5) extraction, completion and provision of metadata, (6) digital analysis of the data, and (7) the option to publish the data and metadata to enable re-use. To overcome this situation, we elaborated several methods that can be combined to transfer data from devices to an ELN and to store these data along with different types of metadata in a FAIR manner. The methods used are designed in a flexible manner to allow its application to various measurement techniques and instruments. In this article, we describe the whole process with the example of cyclic voltammetry measurements.

## Results

Cyclic voltammetry is a powerful technique for the electrochemical analysis of materials which provides important insights into the chemical reactivity and electrochemical properties of the analyzed compounds. Cyclic voltammetry is a standard technique in chemistry,^21–24^ materials sciences^25^ and has also relevance for biological applications.^26,27^ We elaborated a comprehensive example for a “FAIRer” processing of research data using cyclic voltammetry (CV) measurements, as this analytical method combines different challenges that complicate a comprehensive FAIR data-compliant workflow. Solving these challenges and presenting solutions for CV shows how powerful the new developments are and how they can be transferred and applied to other experiment and analysis techniques. The current challenges for FAIR data-compliant cyclic voltammetry data collection are missing standards for the measured data and its metadata, the quite diverse equipment that is used for the measurements, such as different potentiostats and electrodes, and the representation of the results as cyclic graphs with several repetitive measurements that describe one experiment. For transparency and complete reproducibility, the CV measurements should be described using the data generated by the device, including metadata automatically provided by the potentiostat and additional information provided by the user. *E*_g_ the values and parameters that characterize the measurements, but are not recorded in the potentiostat's file, are to be included by users' input. Well-known vendors for cyclic voltammetry equipment are, for example, Metrohm, Gamry and PalmSens. All three manufacturers use their own data format which complicates standardization, readability and reusability of the data and metadata without the use of additional data management software. Usually, the vendor's software supports the export as text file or as CSV, but export formats and metadata included are not standardized amongst the vendors. In some cases, the converted files are also missing important metadata. In order to establish a workflow for FAIR cyclic voltammetry measurements, we elaborated a process that can be applied to the devices and software of the vendors presented herein and can easily be adapted to other vendors' devices. Our process can be described in five parts which together describe how to generate annotated CV measurements in an open and standardized format,^28^ including cases where the original data files are produced by different devices and possibly in different (proprietary) file formats. In the first step, the data is transferred to a digital work or documentation environment which, in our case, is the Chemotion ELN (Fig. 1, step 1). After the data has been received by the ELN, an automated reader can process the data by applying file format and vendor specific profiles to convert the data files to a standardized format. In parallel, the available metadata are extracted from the transferred file and mapped to a predefined metadata scheme. The converted data and metadata are then combined in a BagIt bag (Fig. 1, step 2). The data, now contained in the BagIt bag, can then be plotted, analyzed and annotated as a digital graph in the GUI of the ELN. The analyzed and annotated data and all further documented metadata and their changes are then saved in additional files (Fig. 1, step 3), retaining an unaltered copy of the original data. As the ELN supports the sustainable re-use of the data captured, data and metadata can be transferred to an open access repository or can be downloaded with the full support of data and metadata (Fig. 1, step 4). The described workflow towards FAIR data in CV should be re-useable by other scientists without limitations, therefore all methods and software that were used for the establishment of the workflow are available as open source.

### Step 1: transfer of data from devices to an ELN

Data recorded during a CV experiment using a vendor's software is usually saved locally to the instrument's computer. Fetching the data from that computer involves manual transfer to a physical data storage device, such as a USB drive, or (automatically) copying it to a remote storage location, from which the researcher can access it for further analysis. In order to allow systematic access to data of cyclic voltammetry experiments, we established a routine that automatically transfers the data from the device's PC to the ELN. The routine is explained using a Gamry potentiostat with the vendor's original, unchanged software, “Gamry Framework”. The process was also applied to potentiostats from Metrohm and PalmSens (details described in ESI†). The workflow for such a routine is composed of data recording and storing on the local hard drive, the transfer from the hard drive to a remote storage and the transfer from that storage location to the ELN. In preparation for the data transfer, several points have to be clarified. This includes information on the location the data is saved to on the computer, information on the supported data format(s) and type(s) (is the data stored in folders, single files or as multiple files?), and information on how the data is recorded. The last point is important as some devices write continuously to an initially created file and therefore induce continuously growing file sizes, others create just one data file at the end of the experiment and cache the data until the experiment is complete. In the case of the herein depicted example of a Gamry device, the data file generation is started with the data acquisition and data is written to this file during the experiment. The data file can be assumed to be complete when the data file is closed and no further changes/increase of data content is observed (information on the process for Metrohm and PalmSens can be gained from the ESI†).

### Step 1a: unattended, automatic data transfer to a data exchange location

The transfer of data from a device's computer to a remote location requires a program to monitor the data folder of the instrument's computer. To cover this very special application, a program called Shuttle was developed. The monitoring determines, depending on predetermined criteria, which files or folders are to be transferred at what time to avoid transferring incomplete or unnecessary data. Designing such a monitoring program as Shuttle is challenging as it has to be compatible with many different devices and, therefore, it needs to take into account different scenarios depending on how the data file is produced and saved. Also, the computer's specifications, such as operating system or the processor's architecture, have to be considered. Our solution to the generation of such a flexible monitoring system is the software ShuttleBuilder (see also ESI, Section 2†). Using the ShuttleBuilder, an executable file is created, tailor-made for the environment it will be used in (depending on the device's PC, the network, the remote storage location and available transfer protocols, *etc.*) which can then be set up on every device where such a data transfer is to be implemented. The web-based ShuttleBuilder's GUI allows the administrator to input all the parameters required by the program to transfer the files and generates an executable file as an output, ready to be placed on the device with minimal setup required. The parameters to be defined in the GUI consist of the communication protocol, the operating system it is intended to run on, the source address for the data on the device's computer, the desired target location where the data is to be transferred to and, if required by the remote location, the username and password. Additionally, one can choose what is to be transferred (file, folder, zipped folder) and define necessary delay times to ensure the completeness of the data before they are transferred. This allows integrating systems that amend data files throughout the experiment's runtime, instead of saving completed data files after an experiment has finished. Finally, the ShuttleBuilder GUI instructs the administrator how to set up the executable file on the device's PC and the steps necessary to include the program in the computer's autostart, which will start the program upon booting the operating system. Once set up and running, the program monitors the status of the data and only transfers experiment data files after the data file has been closed, is no longer being written to and hasn't increased in size within the delay time defined when creating the executable.

For the Gamry potentiostat used as an example, a Windows machine with a x64 architecture has to be configured in the ShuttleBuilder. Since the Gamry software produces single files as output, the single file transfer method is chosen and a delay time of 300 seconds ensures that the transfer doesn't take place before the experiment has finished and the data file is complete. Additionally, the local path, where the experiment data is saved to on the device's PC local hard drive and the remote path for the transfer to the ELN, including username and password, are entered into the ShuttleBuilder. Once all parameters have been set, the compilation process can start and once completed, a customized executable instance of the Shuttle with the name efw.exe is then downloaded automatically. Setting up the *efw.exe* file on the device's PC and configuring the operating system to autostart it during booting, is a simple, three-step process (described in detail in the ESI†).

### Step 1b: transfer of data from exchange location to ELN

**Fig. 2:**
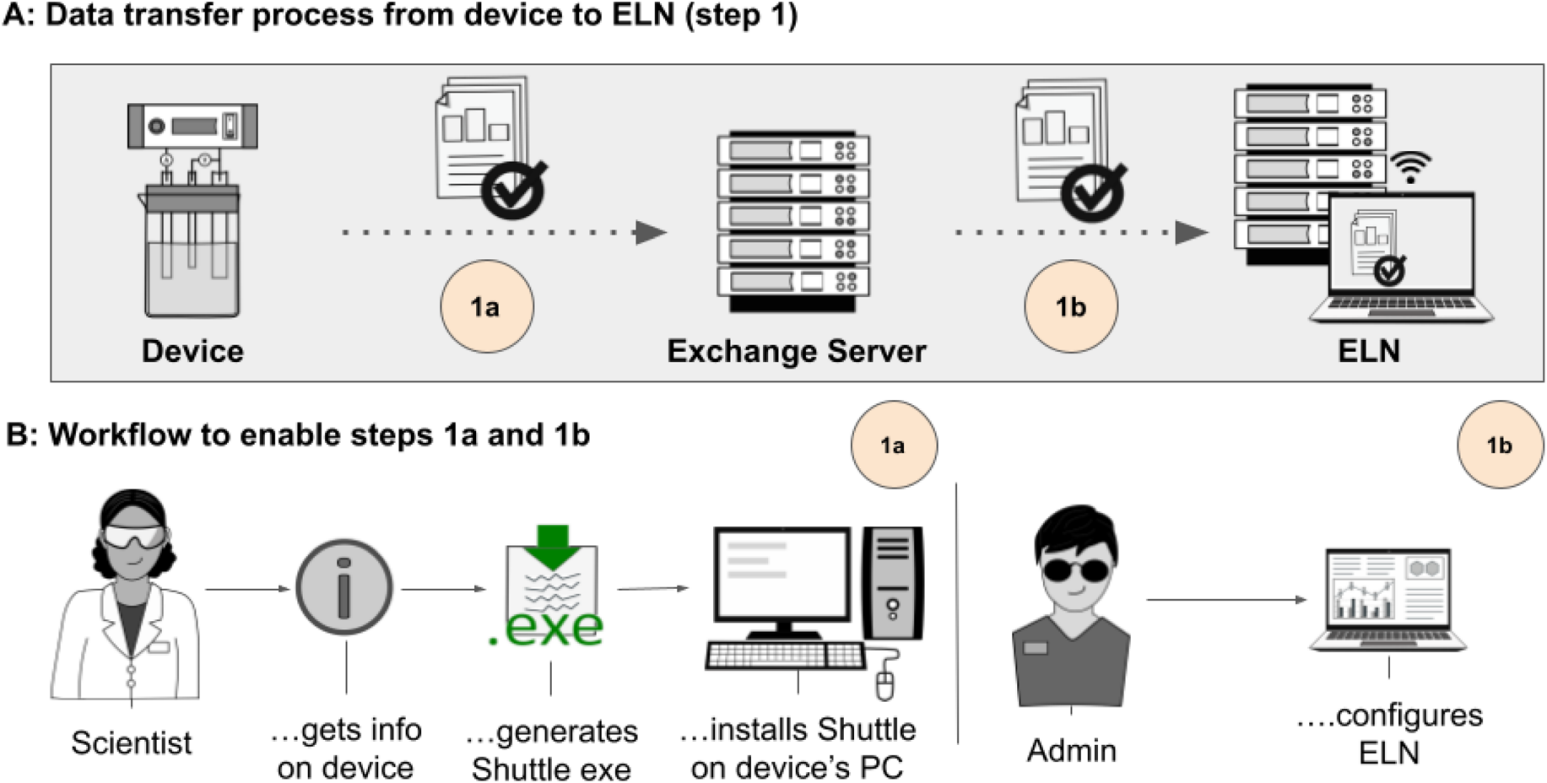
Description of how data are transferred from a device to the ELN server (step 1 of the described workflow to handle data). (A) The transfer consists of two steps which are the mirroring of data to a data exchange server (1a) and the transfer of the mirrored data to the ELN server (1b). (B) Summarizes the actions needed to enable 1a and 1b, both steps are only necessary once during initial device-integration, and run autonomously thereafter. Images used for this figure were generated by C. Henken, KIT-ZML, license: CC BY.

All data that is transferred to a central data exchange server, or other shared location, can be accessed by data management systems if they are configured in a suitable way. In our approach for cyclic voltammetry data, we use Chemotion ELN^17^ as a management system that catches data from the data exchange server. This process was described earlier^18^ and was used without further adaptations (for configuration details in ELN, see ESI†). Applying the ELN routines results in the availability of the data in the inbox of the ELN-UI, where new data can be assigned to the related samples. Saving of the data also includes the choice of a suitable term to describe the type of the measurement. In the case of cyclic voltammetry, the ontology term “cyclic voltammetry (CV)” can be selected from the vocabulary of the Chemical Methods Ontology (CHMO) (Fig. 2).^29^

### Step 2: conversion into open, standardized data

The routine described in step 1 allows to transfer and save the original data file from the measurement, independent of the file format and the content of the data. As FAIR data concepts require the availability of open and standardized data, step 1 needs to be followed by methods for the conversion of the data, in case they are not recorded in open and standardized file formats by the instrument. In our approach, the reading, processing and conversion of the original files is done by *ChemConverter*, a Python-based open source software, which is embedded into the workflows of Chemotion ELN.^30,31^*ChemConverter* works according to a two-step routine: the first time data is obtained from a new device, the data is used to generate a profile that contains all necessary information for the conversion of the obtained file. This profile is stored in *ChemConverter* and is then used for all other data files coming from the device. This process of setting up a routine by an administrator of an ELN allows the users to apply this routine without further needs for configuration or adaptations. The routines of *ChemConverter* include both data and metadata conversion, as described in the following sections.

### Step 2a: data conversion

In order to convert an uploaded file, *ChemConverter* first selects one of a set of implemented generic readers, which are implemented for a set of currently used data formats – and which can be extended by available open source projects in the long run. The reader converts all tables, headers and other metadata that is present in the input file into an internal data structure. The selection and application of a certain profile is triggered by the definition of identifiers that are part of the file's title or content. Once a suitable profile is identified, the data is converted into either one or multiple JCAMP-DX files that, at this stage, include(s) the most important metadata. In the case of cyclic voltammetry, the profiles are configured in such a way, that they provide one JCAMP-DX file per measured cycle (including one anodic and cathodic sweep), resulting in different numbers of converted JCAMP-DX files consisting each of one oxidation and reduction sweep. The JCAMP-DX format^32^ is used because it is supported by IUPAC^33^ and one of the very few standard file formats that are established in chemistry and related domains. Although the format was initially created for storing infrared (IR) spectra,^34^ further specifications for NMR^35^ and other measurement types are available.^36^ The use of JCAMP-DX ensures that the data can be read by open source data viewers such as ChemSpectra^37^ and is therefore a suitable data exchange format for our processes. Nevertheless, the conversion routine can also be adapted to other data formats as output files in the long run, requiring only the support of other writers instead of the JCAMP-DX writer.

### Step 2b: metadata extraction and mapping

**Fig. 3:**
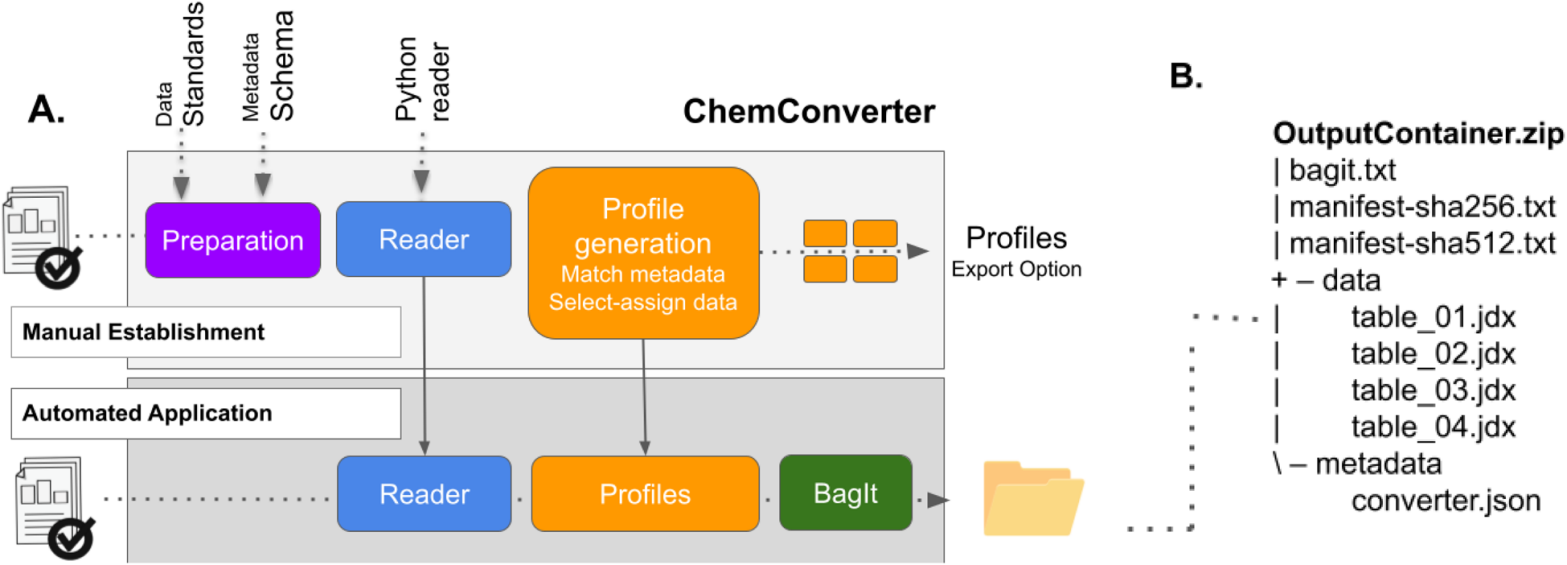
(A) Enabling the extraction and use of data and metadata by implementing a profile for data and metadata mapping. Data is extracted and selected to match the data standard JCAMP-DX and metadata are mapped to a metadata scheme developed by community representatives. Components of *ChemConverter*, given in blue, need manual configuration by users/admins for the generation of a profile the first time a new file type is converted. Once a profile (orange) is generated and stored, further files of the same type (containing the identifier) are converted by the profile, packed into a BagIt bag and are available as a zip file. (B) Outline of the structure of the zip file. Images used for this figure were generated by C. Henken, KIT-ZML, license: CC BY.

In parallel to the conversion of data, *ChemConverter* is also used for the extraction and matching of metadata to predefined metadata schemes. *ChemConverter* can be taught to detect metadata according to diverse rules, using the same profile approach used for the conversion of data. While for data, the JCAMP-DX format (as an available standard) is used, there are currently only a few comprehensive schemes available for metadata and, as far as we know, there is no metadata standard available for cyclic voltammetry. This results in the need to define a metadata scheme that can be used as a common target output for metadata captured from different devices. Recently, the American Chemical Society (ACS) released new guidelines for the description of electrochemical data and, therefore, as part of it, also for voltammetry experiments.^38^ In our approach, the required metadata in this policy was combined with the input gained from different scientific groups as representatives of the cyclic voltammetry user community, yielding a suggestion for a set of metadata that can be used to define a target metadata schema (see details of the metadata scheme in the ESI†).^39^ Following the concept of *ChemConverter* for the conversion of data, a profile for metadata is defined, with an example set of metadata, for each device the first time it is introduced. The profile is defined by the mapping of input metadata to the target metadata scheme that was defined by the community representatives, and the mapping is stored in the profile. These device-specific profiles – which have to be defined just once per device – enable the matching of metadata to the target metadata scheme for further files of the same type. Here again, identifiers that need to be part of all files are used as indicators for the recognition and application of a certain profile (Fig. 3). For cyclic voltammetry, we were successful in the mapping of metadata for data coming from Gamry and PalmSens and the profiles are available for further re-use.^39^ For data coming from Metrohm, the extraction and matching of metadata from the available text file was not possible as described for Gamry and PalmSens yet.‡‡The company is working on the implementation of JCAMP-DX to provide data in an open standard format. To gain some of the most important metadata, such as the scan rate, *ChemConverter* runs a few routines to calculate metadata automatically from the data provided – this allows it to match the most important metadata. After applying the profiles' mapping for Gamry, PalmSens and Metrohm, the extracted and assigned metadata are then added to the JCAMP-DX data file and are additionally stored as a JSON output file.

### BagIt

The data and metadata which are gained as JCAMP-DX and JSON are brought together in the form of a BagIt bag.^40^ If *ChemConverter* is embedded into the Chemotion ELN, the bag is generated as soon as the data from a device is available on the ELN server (and visible in the inbox). The processed data (as original input and converted in a BagIt bag) are visible within the inbox of the ELN and can then be assigned to the samples. BagIt is a well-defined hierarchical file packaging specification.^40^ In our implementation (Fig. 3), the BagIt bag contains a “bagit.txt” file, a data/payload subdirectory and two manifest tag files (sha256 and sha512) that include a checksum for each file in the payload directory. In case of the CV output, the payload data directory contains different converted files in JCAMP-DX format, each the result of an extracted and converted CV cycle from a single input file. Additionally, the bag contains a directory named metadata, which consists of a JSON file with the sum of all extracted metadata of the whole CV experiment.

### Step 3: visualization of data and analysis with *ChemSpectra*

**Fig. 4:**
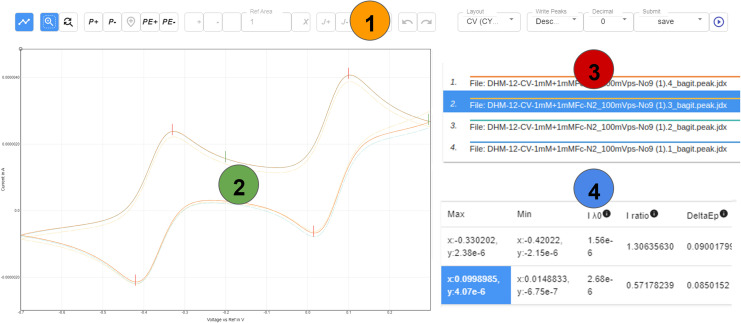
General schematic overview of the different information/action panels of a CV layout in *ChemSpectra*. Additional metadata such as scan rate, solvent and others are not described here and are part of the metadata section (see ESI†). (1) Action panel that allows to select and switch between different actions (*e.g.* peak picking); (2) the plotting area shows all cycles and highlights the one selected in area (3); (3) the graph selection area can be used to select a single cycle (out of different ones given in the plot); (4) for a selected sweep, the picked minima and maxima are summarized in a table form and used for the calculation of *i*_pa_/*i*_pc_, *E*_1/2_ and Δ*E*_p_. The different panels (1–4) were selected from a screenshot of *ChemSpectra* and the ratio of size was changed for a clearer representation.

According to the concept of Chemotion ELN, the bag, but also the original file that was generated by the device, should be assigned to the corresponding sample representation in the ELN. Both are then part of the so-called analysis description of a sample and can be used for further analysis, can be retrieved per download and can be transferred to other systems such as repositories. The manufacturers of potentiostats usually deliver advanced software for evaluation and visualization of the measurements. Therefore, the analysis of cyclic voltammetry measurements is usually done using the vendors' software. Unfortunately, the analysis software of most of the vendors is proprietary and neither available nor suitable for an embedding into open source developments, such as Chemotion ELN. To overcome limitations in the use and analysis of the CV data, the data viewer *ChemSpectra*,^37^ an open source, browser-based tool for data visualization and evaluation, was adapted to the use case of CV.^41^*ChemSpectra* can handle either single spectra, provided in JCAMP-DX format or multiple spectra files in a BagIt bag. Multiple files containing BagIt bags are processed by *ChemSpectra* to show each spectra file as a separate graph, or to show different combinations of selected files in one graphical summary. *ChemSpectra* was systematically extended for CV measurements to enable the work with multiple cyclic voltammograms in one plot. This involved adapting the UI to allow the selection of individual curves from a set of curves, and advanced identification options for anodic and cathodic peaks had to be developed. *ChemSpectra* further allows the calculation and assignment of some standardized key properties to describe and compare different curves of CV experiments. The most common properties are the correction value of the capacitive background current *I*_λ0_ (sometimes also *i*_sp0,_ currently determined graphically by the user), the current ratio *i*_pa_/*i*_pc_ of a given redox-pair,^42^ the half wave potential (*E*_1/2_) and the peak separation between the anodic and cathodic peak (Δ*E*_p_) (Fig. 4). The selection of peaks *via* peak picking and the calculations depending on the choice of peaks are summarized in the information section of the UI in *ChemSpectra*. Additionally, the results can be downloaded as a table CSV file to allow the fast integration of the results into publications and reports (see example in ESI†). To further support the scientists in the standardized documentation of their analyzed data in textual form, the software additionally supports the summary of the results in the form of an inline notation that can be directly used for reporting purposes.^43^

### Step 4: summarized metadata scheme and data file output

**Fig. 5:**
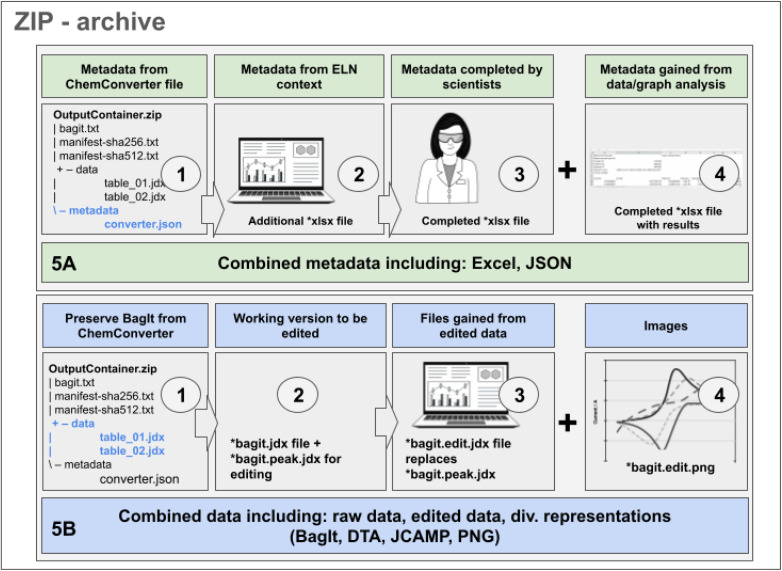
Schematic summary of the components contributing to metadata and data describing a CV experiment with respect to information coming from the device, automated processes, manual adaptation and completions. (A) Components of a typical metadata collection; (B) components of a typical dataset for CV measurements and processing in Chemotion; images used for this figure were generated by C. Henken, KIT-ZML, license: CC BY.

As in most of the cases the metadata of the measurement's data file do not fully cover all details, the extracted metadata should be completed with contextual information that is available in the ELN (such as the sample name and identifiers that were used for the measurement) or with manually added information. The option to complement extracted data with additional information is of high importance in the case of cyclic voltammetry, as the information captured from the potentiostat is only one part of the experiment's description. The information gained from the file needs to be completed by data describing the setup, such as the type of electrodes, the measurement's conditions, and other parameters of the measurement (a list of all metadata is given in the ESI†). In summary, a first part of the metadata can be obtained from the extraction of the data file by *ChemConverter* as depicted in Fig. 3 (Fig. 5A, part 1), a second part of the metadata, such as information on the sample and the author of the data, is available from the ELN-database (Fig. 5A, part 2) and a third part of the metadata is provided manually by completion of the ELN forms (Fig. 5A, part 3). In case the data files of the measurement were analyzed in *ChemSpectra*, a set of metadata including the key values of the analysis is generated as the fourth part of the metadata and is also included in the download option for metadata (Fig. 5A, part 4). After completion of the metadata, comprehensive information is available and can be downloaded as an XLSX file to ensure that all the combined metadata is available in an easily human-readable form. For CV experiments, a typical metadata table file consists of at least three sheets. One is reserved for explanations and definitions of the file and its labels, a second one gives all metadata gained from steps 1–3, and a third one (or more sheets) are added to give the graph-related analysis data (*x*CV curves = 2 + *x* sheets) (Fig. 5, A).

The data generated by devices such as the CV potentiostats and processed in a BagIt bag (Fig. 3) undergo several changes during the further research data life cycle. These changes have to be amended – similar to the completion of metadata – to the available dataset. While working with the data, the original bag (Fig. 5B, part 1, including the JCAMP-DX as obtained from *ChemConverter*) is preserved. One or multiple working versions of the data – depending on the number of CV cycles/files – available as *.bagit.jdx are created by *ChemSpectra* and changes made to the data through the automation routine, *e.g.* through automated analysis tools, are saved automatically to one or multiple additional file(s) labeled *.*bagit.peak.jdx* (Fig. 5B, part 2). Files that are changed based on manual actions, such as manual peak picking with the UI, are renamed to .*bagit.edit.jdx* (Fig. 5B, part 3) while keeping the original data as a separate block inside the JCAMP file (for the complete workflow of *ChemSpectras* front- and backend see ESI†). Additionally, files for the representation of the data as an image are generated based on the edited data and are available as *.*bagit.edit.png* (Fig. 5B, part 4). As CV data in *ChemSpectra* are handled in a way that allows the generation and tracking of one data file per curve that is measured, the data processing and saving routines apply to all curves/files individually, allowing the generation of a fine granular summary and re-use of the data.

Typical re-use scenarios, such as the download for sharing to enable new research, would require the availability of data and metadata. Therefore, our CV approach to FAIR data includes a collection of the results from the processes described in Fig. 5A and B. A combined ZIP archive includes all metadata generated by different sources and processes. It also combines all data obtained as raw, automatically selected and manually edited modifications in different representations (Table 1).

**Table 1:** Summary of all obtained metadata and data for a raw dataset after passing the conversion and editing workflow of Fig. 5. The combined (meta)data for all curves and the single curves can be downloaded in the form of a zip-archive. The example given here consists of only one CV curve, for each additional curve, the files of type b (see table) are repeated

Name convention	Ending, type	Content	Explanation
**Zip content (type a): files covering all content – all CV curves**			
dataset_description	*.txt, Text	Metadata	Main metadata including a list of all files included to the zip archive
name	*.DTA, DTA	(meta)data	Exemplarily taken original data file als gained from the potentiostat, raw data for archiving purposes
name	*.zip, BagIt	(meta)data	BagIt bag as obtained from the conversion routine of the converter (applied to DTA raw data), including *e.g.* a file in JCAMP-DX format (data) and JSON (metadata). The bag is included in the zip for archiving purposes
shortname	*.xlsx, excel sheet	(meta)data	Summary of all metadata and results for the overall dataset and single curves in one multi-sheet containing excel. Edited file and/or for editing purposes
name.combined	*.png, image	Data	Picture generated automatically as a summary of all curves (processed from original data). File for data preview purposes
name.new_combined	*png, image	Data	Picture generated after selection of relevant curve(s) (processed from edited data for publication purposes). File for data preview purposes

**Zip content (of type b): files covering parts of the original measurement – stored for each CV curve**			
name-curve_bagit	*.jdx, JDX	Data	Data of one curve processed to the standardized file format JCAMP-DX from the BagIt bag. Edited file and/or for editing purposes
name-curve_bagit.peak	*.jdx, JDX	Data	Data of one curve in JCAMP-DX format. The file is gained from the original BagIt bag including automatically picked data points. File is not present in all archives – as it is not generated in all cases and is replaced by _bagit.peak file if manual editing occurs
name-curve_bagit.edit	*.jdx, JDX	Data	Edited data of one curve in JCAMP-DX format. The file is gained from the original BagIt bag and replaces existing _bagit.peak files. Edited file and/or for editing purposes
name-curve_bagit.edit	*.png, PNG	Data	Picture giving a data plot of one curve. File for data preview purposes
name-curve_bagit.edit	*.csv, CSV	Metadata	Metadata of one curve as a reduced information of the overall information in the *xlsx file. Edited file and/or for editing purposes (ESI, Fig. S8)

### Deposition of the data in a repository

**Fig. 6:**
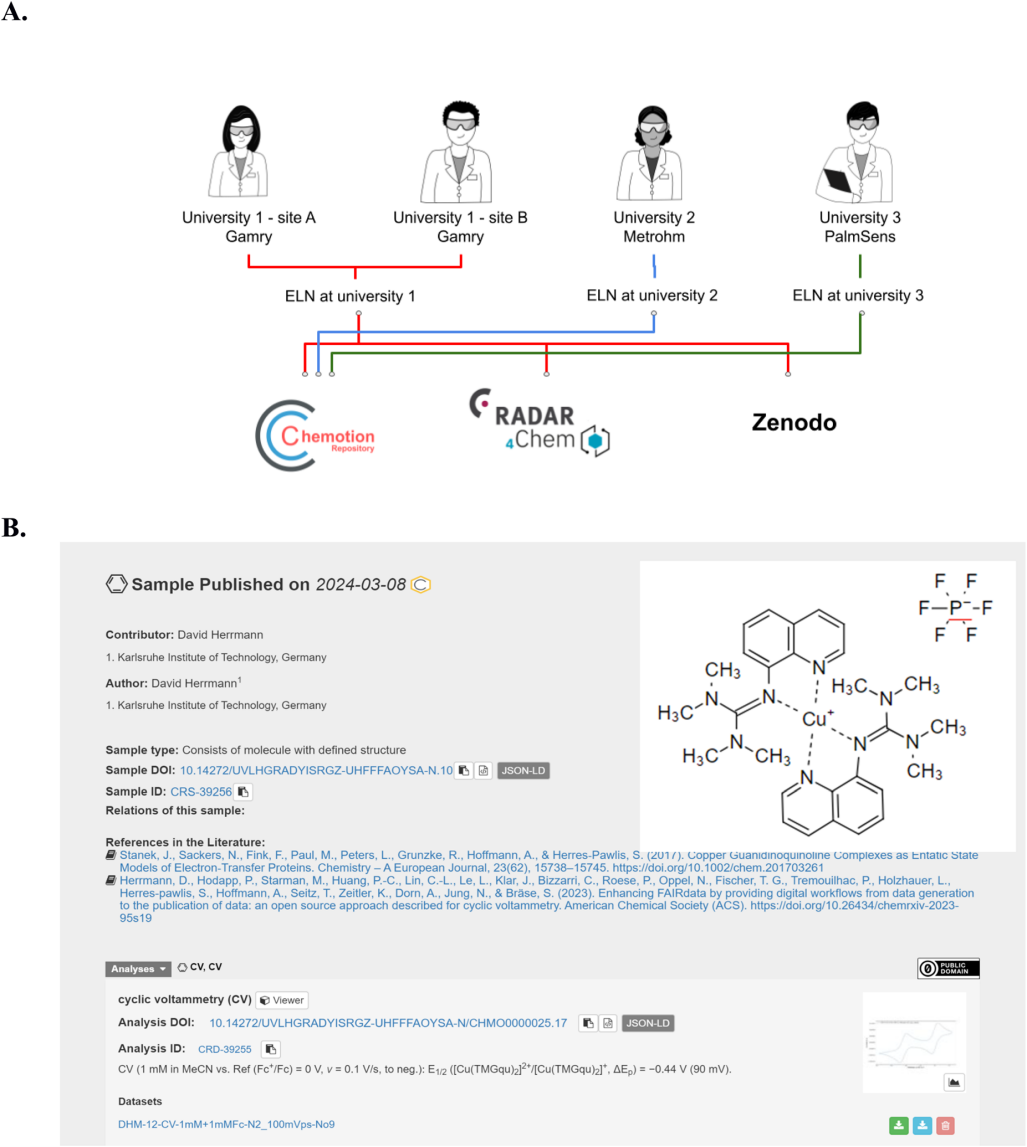
(A) Summary of the submission of CV measurement data to different repositories: University 1 (KIT) contributed with datasets from Gamry devices running at two different sites having access to the same ELN instance. University 2 (RWTH Aachen University) generated the data using a Metrohm device. University 3 (Leipzig University) applied a PalmSens device. All three submitted data to the Chemotion repository, while KIT submitted data to all three repositories (Chemotion Repository, Zenodo and RADAR4Chem, see ESI Section 8†). This allows for a comparison of data submission workflows to three distinct repositories. Images used for this figure were generated by C. Henken, KIT-ZML, license: CC BY. (B) Example for a representation of CV measurement data generated with a Gamry device, as published in the Chemotion repository. The content of the figure was gained from a screenshot (see ESI†) with rearrangement for a better visibility.

All of the steps 1–4 described above deal with the preparation and handling of data on site. The information is visible only to the owner of the experiments and optionally to colleagues the experiments were shared with. Making the data findable and accessible to the community requires the submission to a research data repository. Using the example of CV data, we showed that all information that was directly obtained from the device, as well as additional data and metadata obtained during the analysis and documentation steps, can be published in repositories to make the information available to others. Fig. 6A summarizes the options that were showcased in this work: data that was gained in different groups at different sites (KIT Helmholtz research area, KIT university area, RWTH Aachen University and Leipzig University) were published through different workflows in Chemotion repository, RADAR4Chem, and/or Zenodo. The different repositories were selected to support a comparison of the data after their submission and to facilitate a selection of the right repository according to the uers' preferences. All three groups that established the workflow from step 1–4 used either the Cu(i)-complex [Cu(TMGqu)_2_]PF_6_ (see Fig. 6A) or Cu(dmp)_2_BF_4_ (see ESI†) or both as a reference example for their CV experiments. This allows for the comparison of the data gained from different devices. The differences of choosing either Chemotion, RADAR4Chem or Zenodo are primarily influenced by the submission workflows and secondarily by the way the data are represented. As Chemotion and RADAR4Chem are supported by the herein used ELN software in a way that data can be transferred from the ELN to the repository, the provision of data to Chemotion and RADAR4Chem is straightforward and needs only a few clicks to publish the prepared data as depicted in Fig. 5. Worth mentioning is that the analytical data can only be disclosed in combination with the sample that was used to prepare the data – in this case Cu(dmp)_2_BF_4_ and/or [Cu(TMGqu)_2_]PF_6_. Scientists that chose repositories that are not directly connected to the ELN can also easily publish the obtained results. The publication in Zenodo or any other repository can be achieved by downloading the CV measurements from Chemotion ELN in the form of a zip file (content described in Fig. 5) and a subsequent data submission at the repository of the researcher's choice. A summary of information, as it is presented in different repositories, is provided in the ESI.†

## Conclusion

This work describes methods for a “FAIRer” handling of research data using cyclic voltammetry as an example. The suggested procedure includes concepts, software and infrastructure that can be used to support all stages of the data life cycle – from the initial collection of data to the publication of data and metadata in repositories. Our workflow includes: digital data acquisition, the data transfer from devices to an ELN, the assignment of the data to the experiment/sample, the processing of the data into a readable and standardized file format, the extraction, completion and provision of metadata, the digital analysis of the data, and the option to allow for re-use of the full set of data and metadata in other systems by data publishing in repositories. We demonstrated the potential of the described processes to improve the transparency, reproducibility and re-usability of cyclic voltammetry (CV) data. The described approach provides a harmonized set of data and metadata, even if the measurements are performed using devices from different vendors and/or at different locations, due to the application of standard data formats and metadata schemes. The approach was finalized by transferring the data and metadata, obtained by different scientific groups, from the ELN Chemotion to different repositories. The methods described for cyclic voltammetry can be adapted and used for other analytic characterization, and data collections, hence providing a simplified way to integrate new methods for digitalized research and FAIR data management.

## Abbreviations

CV: Cyclic Voltammetry
DB: Database
DMP: 2,9-Dimethyl-1,10-phenanthroline
ELN: Electronic Laboratory Notebook
FAIR: Findable, Accessible, Interoperable, Reusable
GUI: Graphical User Interface
LIMS: Laboratory Information and Management System
TMGqu: 1,1,3,3-Tetramethyl-2-(quinolin-8-yl)guanidine
UI: User Interface

## Data availability

All software that is used to gain the work described is provided as an open source on GitHub with some parts additionally tagged under version control on Zenodo. In detail, the following components were used (each assigned to a certain step in Fig. 1 to explain its role): step 1: ShuttleBuilder software (https://doi.org/10.5281/zenodo.11209067), step 2: Chemotion ELN version 1.9.3 (https://doi.org/10.5281/zenodo.11237613), including generic dataset functionality to generate and apply metadata schemes for data files, converter software to convert different file types into JCAMP-DX and match metadata (https://doi.org/10.5281/zenodo.8033807, https://github.com/ComPlat/chemotion-converter-app, https://github.com/ComPlat/chemotion-converter-client), step 3: embedded data viewing software *ChemSpectra* (*ChemSpectra* app https://github.com/ComPlat/chem-spectra-app and ReactSpectra editor https://github.com/ComPlat/react-spectra-editor), step 4: software ELN version 1.8.0 or later (see above) and chemotion repository (https://doi.org/10.5281/zenodo.8093570) can be used. In addition to the source code, we provide the full documentation for the use of our systems such as the Chemotion ELN and Chemotion repository (https://www.chemotion.net/docs/repo), the data used for the development of the herein described data conversion and metadata mapping (https://doi.org/10.5281/zenodo.12827203) and additional information on how to install and configure the herein described components for CV but also other measurement types beyond CV (https://www.chemotion.net/docs/services/chemconverter, https://www.chemotion.net/docs/eln/devices). The examples that were depicted in this work were published as research data in the repositories Chemotion, RADAR4Chem and Zenodo. The example depicted in Fig. 6B was deposited with the DOI https://dx.doi.org/10.14272/UVLHGRADYISRGZ-UHFFFAOYSA-N.10, all other repository entries are described in the ESI Section 8† where also the respective DOIs are given.

## Author contributions

NJ and SB contributed to the conceptual work of this project and provided the first draft of the manuscript. MS, JK, LL, CLL, PCH, and PT designed the technical processes, developed and adapted software to enable the processes described in this work. DH collected use cases, coordinated all partners, proposed metadata schemes and layouts for the technique CV. PH and MS enabled the integration of devices into the digital workflow of the ELN. CB, PR, SHP and AH and TGF acted as early adopters of the herein presented methods and contributed with ideas and suggestions for the improvement of the overall process and the methods in detail. TGF implemented the integration of a PalmSens CV instrument in the Chemotion ELN instance at Leipzig University and reviewed and edited the manuscript. AD, TGF, NO, DH and TS conducted the experiments cited in this work as reference examples and published the data as reference data in different repositories. LH and KZ contributed with conceptual ideas.

## Conflicts of interest

The authors declare no competing interests.

## Supplementary Material

SC-OLF-D4SC08620A-s001
