# Supplementary material for: Enhancing FAIRdata by providing digital workflows from data generation to the publication of data: an open source approach described for cyclic voltammetry
Source: Chem Sci. 2025 Feb 5;16(10):4430–41. doi: 10.1039/d4sc08620a (PMC11796049; doi:10.1039/d4sc08620a)
Supplement: SC-016-D4SC08620A-s001 [file SC-016-D4SC08620A-s001.pdf]

Supplementary Information (SI) for Chemical Science.
This journal is © The Royal Society of Chemistry 2025

Enhancing FAIRdata by providing digital workflows from data generation to the publication of data: an open source approach described for cyclic voltammetry

David Herrmann[a], Patrick Hodapp[b], Martin Starman[a], Pei-chi Huang[a], Chia-Lin Lin[a], Lan B. Q. Le[a], Tillmann G. Fischer[f], Claudia Bizzarri[d], Philipp Röse[e], Niklas Oppel[e], Jochen Klar[c], Pierre Tremouilhac[a], Laura Holzhauer[a], Sonja Herres-Pawlis[g], Alexander Hoffmann[g], Tobias Seitz[g], Alrik Dorn[h], Kirsten Zeitler[h], Nicole Jung[a,i], Stefan Bräse*[a,d]*

Email: nicole.jung@kit.edu; stefan.braese@kit.edu

P
A

Content

1 General definitions and explanations of included tools

2 Details and explanation for enabling the transfer of data from potentiostats to an ELN

2.1 Option 1, Step 1a: Unattended, automatic data transfer to a data exchange location with ShuttleBuilder

2.1.1 How to get a Shuttle by using ShuttleBuilder

2.1.2 How to use the Shuttle with a potentiostat's PC

2.2 Option 1, Step 1b. Transfer of data from exchange location to ELN

2.3 Option 2, Data transfer from device using rsync and rrsync

2.3.1 Step 1a: Data transfer to the device to a large data storage with rrsync

2.3.2 Step 1b: Transfer of new CV data from large data storage to the folder watched by Chemotion ELN's Data Collector with rsync

2.3.3 Step 1c: Sync of CV data on large data storage and ELN server

3 Data and Metadata conversion: generating the converter's profile

3.1 Step 2a: Data conversion with ChemConverter in detail

3.1 Step 2b: Metadata mapping with ChemConverter in detail

Example for downloaded table format

4 BagIt Implementation

5 Special features for Cyclic Voltammetry in ChemSpectra

6 ChemSpectra Workflow

7 Inline Notation

8 Representation of data in different repositories

9 References

P
A

1 General definitions and explanations of included tools

Chemotion-ELN[1]**:** *Chemotion ELN* is an open source software that is used in this work to execute the suggested workflow including different software components and methods. It includes different components that facilitate the work with scientific data, e.g. the *Data Collector*, *ChemSpectra*, and routines to store data and to export them.

Link to the code resource: https://github.com/ComPlat/chemotion_ELN; Link to documentation: https://chemotion.net/docs/eln

Shuttle: *Shuttle* is a software that can be executed on a computer of scientific instruments, allowing the transfer of data from the device's computer to a remote location. *Shuttle* monitors the data folder of the instrument's computer. The monitoring determines, depending on predetermined criteria, which files or folders are to be transferred at what time to avoid transferring incomplete or unnecessary data. As *Shuttle* needs to be compatible with many different devices it is individually configurable per device and environment. The configuration is done via the GUI of ShuttleBuilder.

Link to the code resource: https://github.com/ComPlat/shuttle; Link to documentation: https://chemotion.net/docs/eln/devices/configurations/data_transfer

ShuttleBuilder: *ShuttleBuilder* is a software that can be used to build an executable *Shuttle* file. *ShuttleBuilder* is currently offered as a self-hostable service to allow the straightforward generation of *Shuttles* which are used for transferring data from devices to another location. The *ShuttleBuilder* allows to generate a tailor-made software (*Shuttle*) for the device and environment it will be used in (depending on the device's PC, the network, the remote storage location and available transfer protocols, etc.) which can then be set up on every device where such a data

transfer is to be implemented. The web-based *ShuttleBuilder*'s GUI allows the administrator to input all the parameters required by the program to transfer the files and generates an executable file as an output, ready to be placed on the device with no further installation required.

Link to the code resource and documentation: https://github.com/ComPlat/shuttlebuilder; Link to documentation: https://chemotion.net/docs/eln/devices/configurations/data_transfer

Data Collector[2]**:** The *Data Collector* is part of *Chemotion ELN* and can be configured via the ELN Admin UI. The *Data Collector* feature imports data automatically from a folder which is monitored. The data is made available to the users in their inbox of the ELN following file or folder naming conventions. The data collector may collect folders or files, depending on how it is configured and whether analytical data is provided as folders or files. Alternatively, the *Data Collector* may not watch a local folder but fetch data from another storage via SFTP.

Link to the code resource: is part of the Chemotion ELN source code (see above); Link to the documentation: https://chemotion.net/docs/eln/devices/configurations/local_collector

ChemSpectra:[3] *ChemSpectra* is a software that can be used as stand alone software or integrated into other systems. It can be used to view and analyze data files from analytical instruments such as NMR devices, mass spectrometers, or potentiostats (in the work described here).

Link to the code resource: App: https://github.com/ComPlat/chem-spectra-app and client for ELN: https://github.com/ComPlat/react-spectra-editor; Link to the documentation: https://www.chemotion.net/docs/repo

ChemConverter: *ChemConverter* converts different file formats and types from integrated devices to common and well known formats such as JCAMP-DX and JSON. It extracts columns

for x,y- data visualization and metadata and maps them to predefined structure (xy data and xy points) and metadata schemes (metadata). Currently, the *ChemConverter* can handle most text readable files like TXT, CSV or DTA, as long as the content is properly structured. Additionally, developers can create their own readers, if there is no reader available already. *ChemConverter* runs as a background process of *Chemotion ELN*, when a user or device (via *Data Collector*) provides analytical data, but may also be used as a standalone application.

Link to the code resource: https://github.com/ComPlat/chemotion-converter-app; Link to the documentation: https://www.chemotion.net/docs/services/chemconverter

Chemotion Repository:[4] The Chemotion repository is a research data repository, available as a service hosted by the Karlsruhe Institute of Technology that allows chemists and other scientists to share data from experimental chemical (molecular) research in a reusable and traceable manner, incorporating domain-specific standards. Chemotion focuses on experimental synthesis and the associated protocols, the representation of chemical compounds and their properties, and their characterization using analytical data. The chemotion repository was used (besides RADAR4Chem and Zenodo) in this work to demonstrate how CV data can be made publicly available through research data repositories.

Link to the code resource: https://github.com/ComPlat/chemotion_REPO/ ; Link to the documentation: https://www.chemotion.net/docs/repo

2 Details and explanation for enabling the transfer of data from potentiostats to an ELN

2.1 Option 1, Step 1a: Unattended, automatic data transfer to a data exchange location with ShuttleBuilder

2.1.1 How to get a-Shuttle by using ShuttleBuilder

General information: First, the administrator can choose from two different protocols, either SFTP (based on SSH) or WebDAV (based on HTTP(S)), and needs to enter the operating system the program is intended for, either Ubuntu x64, Windows x64 or Windows with a x32 bit architecture. Given that the Shuttle project is coded in the GO programming language, it offers the flexibility to employ SSH-based protocols across operating systems that may not inherently support such protocols. However, it's worth noting that on Windows XP-based computers, the GO SSH functionality isn't supported. In such cases, it becomes necessary to install WinSCP, which is conveniently provided by the ShuttleBuilder. The source address for the data on the device's computer must be given, and the desired target location where the data is to be transferred to must also be entered. If required by the target storage location, the username and password can also be specified. Finally, the administrator can choose between three transfer methods, either transferring only individual files or transferring an entire folder newly created by the software. A third possibility is to zip folders before their transfer. The last option the administrator has to specify is a delay time that the program will wait for before transferring them to the target. This allows integrating systems that amend data files throughout the experiment's runtime, instead of saving completed data files after an experiment has finished. The program will additionally monitor the status of the data and only transfer it after the data file has been closed and is no longer being

written to. Finally, the builder GUI instructs the administrator how to install the program on the PC and the steps necessary to instruct the PC to autostart the program after booting the operating system and running the program in the background, as it requires no further user or administrator input.

The Gamry example: In the following section, the options that need to be set in the Builder for a Gamry device are introduced (see also Figure S1).

The computer managing the Gamry device is a modern Windows machine with an x64 architecture. Since the Gamry potentiostat produces single files as output, the transfer method can be set accordingly and as transfer protocol SFTP is used. Additionally to the protocol, architecture and transfer information, a wait time of 300 sec. and the required path information are entered into the *ShuttleBuilder*. The download of the *Shuttle* that can now be executed in the browser-based user interface of the *ShuttleBuilder* initializes the compile process at first. Once the compilation process is successfully completed, the *Shuttle* as a file called *efw.exe* is automatically downloaded.

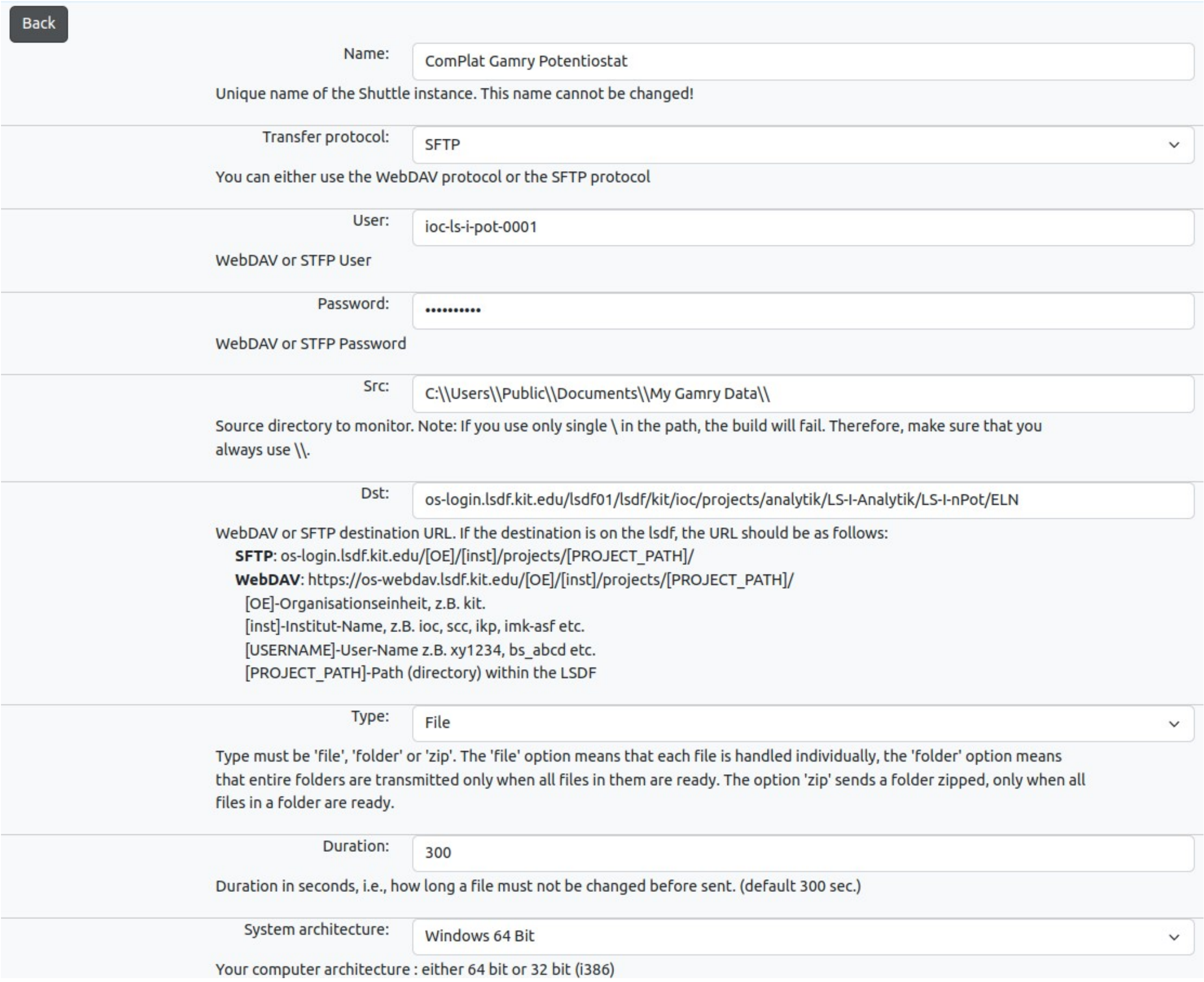

Figure S1: The GUI of the transfer program's web-based *ShuttleBuilder* allows the administrator to input all parameters for the transfer of data from a device's PC to a target location for ELN import.

2.1.2 How to use the Shuttle with a potentiostat's PC

Next it is explained how to complete the installation on the device`s PC. Beforehand, however, it is worth mentioning that access rights to this device are restricted for the standard user. Hence, the installation has to be completed by an authorized system admin. The installation itself is simple and can be described in three steps. Firstly, a directory "C:\Program Files\eln_exporter" has to be

made. Secondly, the downloaded *efw.exe* has to be copied into "C:\Program Files\file_exporter". Finally, a *file_exporter_task.vbs* (Visual Basic script) has to be created in the startup directory of the device. This file_exporter_task.vbs can be downloaded from the builder and looks as follows:

```
Dim WinScriptHost
Set WinScriptHost = CreateObject("WScript.Shell")
WinScriptHost.Run Chr(34) & "C:\Program Files\file_exporter\efw.exe" & Chr(34), 0
Set WinScriptHost = Nothing
```

This completes the installation. After the device is restarted the file_exporter_task.vbs will automatically trigger the monitoring and transferring of the Gamy output files.

2.1.3 Dependency statement for the Shuttle

The Shuttle is developed in the Go programming language. At the time of publication Shuttle supports the latest Go version 1.23. Go is a compiled programming language that offers a simple interface for cross-compilation. This enables the Shuttle to be compiled for almost all devices with minimal effort. The ShuttleBuilder currently supports all Linux systems, as well as Windows 10 and Windows 11. Even Windows XP and Windows 7 on i386 processors are supported. If support for another system is required, the Shuttle can be compiled manually or it can be extended in consultation with the development team.

2.2 Option 1, Step 1b. Transfer of data from exchange location to ELN

The configuration is done on the ELN side as described in the documentation (https://chemotion.net/docs/eln/install_configure/configuration?_highlight=datacollecto#data-collector-settings)

The system of the exchange location allows for sftp access. The ELN data collection is then configured accordingly using key authentication through the Chemotion ELN Admin interface.

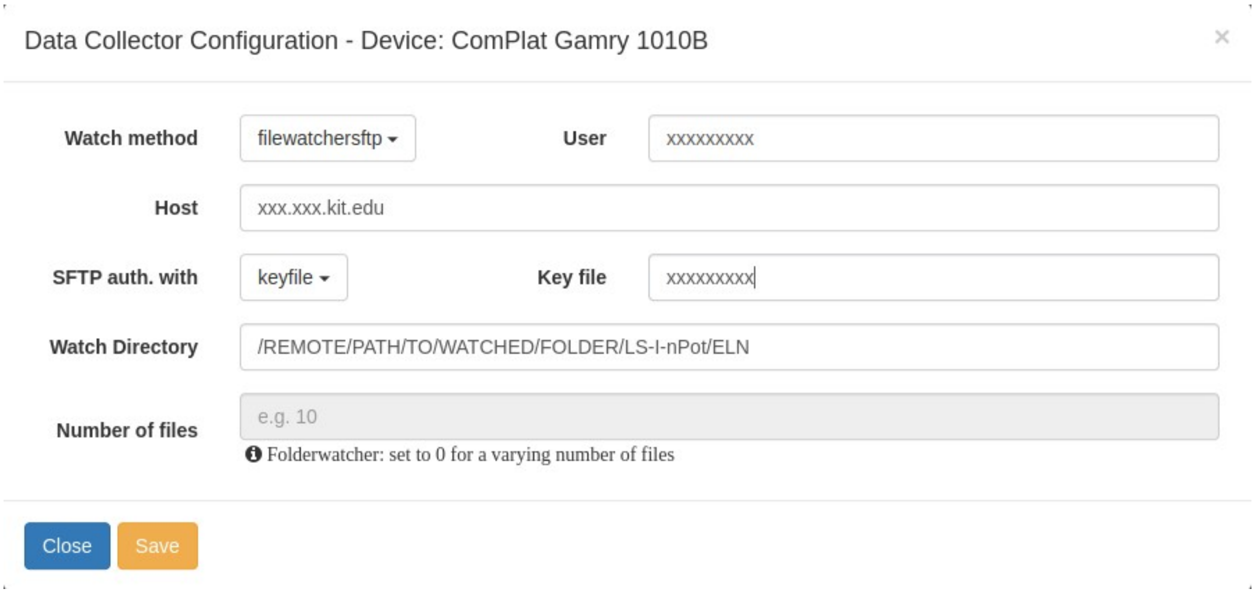

Figure S2: Configuration of the Data Collector in ELN

File collection is configured in the config/datacollectors.yml file to run every few minutes using a cron syntax. In our case:

```
production:
  :services:
    - :name: 'filewatchersftp'

      :cron: '03,13,23,33,43,53 5-22 * * *'

 …

 # Dir of ssh keys for data collection over sftp

 :keydir:                                   "config/data_collector_keys"
```

2.3 Option 2, Data transfer from device using rsync and rrsync

At Leipzig University, rsync and rrsync was used to transfer data. The machine controlling the CV device runs Windows 10 22H2 with OpenSSH for Windows with PowerShell as default shell and WSL (version 2.0.9.0, Kernel 5.15.133.1-1, as of 2023-11-17) with OpenSSH with bash as default shell (as of 2023-11-17). The following data transfer routine takes a maximum of three minutes

from the start of the data transfer on the computer controlling the device until the data is available in the user's ELN inbox. The overall process is visualized in Figure S3.

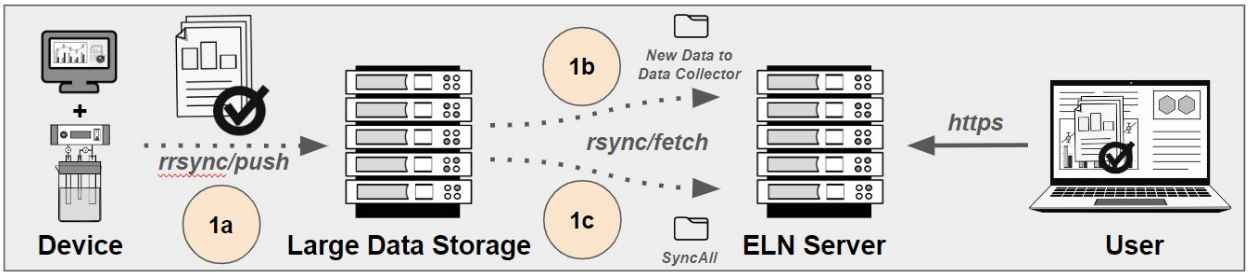

Figure S3: Data transfer process from device to ELN with rsync and rrsync used at Leipzig University.

2.3.1 Step 1a: Data transfer to the device to a large data storage with rrsync

Users save their analytical CV data (in the case of PalmSens EmStat3 with PSTrace software) as .pssession files on the local hard drive of the machine controlling the CV device in a designated folder, but may and tend to choose their own (sub)folder structure, hence, data is aggregated first and then pushed to the large data storage with rsync/rrsync.

PalmSens PSTrace software automatically saves a file for each run in a “autosave” folder, but a measurement including many runs is saved by the user at the end of the measurement including many runs i.e. the software does not continuously save data to a file as this is the case with the Gamry software.

The data transfer is initiated by the user via a shortcut on the desktop which runs a powershell script to start WSL, runs “find”, pipes the list of files to rsync to push the CV data to a large data storage via SSH with a passwordless key file for authentication. In order to restrict any other use than rsync and access to any other folder than the designated folder for CV data on the large data storage with that keyfile, rrsync was employed, see the rrsync man page https://download.samba.org/pub/rsync/rrsync.1.

This is the slowest step of the data transfer routine, as “find” is inherently slow. The aggregation of all .pssessions and subsequent rsync took 3 sec as of 2023-12-07.

2.3.2 Step 1b: Transfer of new CV data from large data storage to the folder watched by Chemotion ELN’s Data Collector with rsync

The server hosting Chemotion ELN holds the second copy of all research data (*cf.* 3-2-1-0 rule). In order to only copy new CV data from the large data storage to the local folder watched by Chemotion ELN’s *Data Collector*, the ELN server fetches only the new CV data from the large data storage by comparing the folder on the ELN server with CV data on the large data storage via rsync’s --compare-dest option. The new CV data rsynced to the folder watched by Chemotion ELN’s Data Collector is then ingested by Chemotion ELN and made available to the ELN users in their inboxes.

This is automated via cron and each run takes less than a second (as of 2023-12-07).

File collection is configured in the ADM UI as well as the config/datacollectors.yml as filewatcherlocal which also specifies the time between each run and the local folder:

```
production:
  :services:
    - :name: "filewatcherlocal"
      :every: 2 #minutes
  :localcollectors:
    - :path: "/chemotion/datacollector"
```

2.3.3 Step 1c: Sync of CV data on large data storage and ELN server

Finally the folder holding all .pssessions files on the ELN server is synced with the corresponding folder on the large data storage, so that both servers hold a separate and identical copy of all CV data.

This is also automated via cron and each run takes less than a second (as of 2023-12-07).

P
A

3 Data and Metadata conversion: generating the converter's profile

The profile consists of specific rules defined with the help of drop-down menus inside the GUI and must be predefined for each measurement method and device at least once. The decision on the used profile and the mode of action is made by the admin of the system who configures both for the single ELN instance. Table 1 lists the different vendors and possible identifiers.

Table 1: Conversion of data

Manufacturer	Output File	possible identifier	additional notes
Gamry	.DTA text file	● DTA file extension<br>● header contains "Cyclic"	
PalmSens	.pssession JSON file	● pssession file extension<br>● value of "title" key is "Cyclic Voltammetry"	exports in other formats (e.g.xlxs) possible with PalmSens PSTrace software but with the loss of many metadata
Metrohm	simple csv formatted .txt file	● titles of columns<br>○ e.g. "Potential applied (V)" and "Scan"	no metadata included

3.1 Step 2a: Data conversion with ChemConverter in detail

Cyclic voltammetry comes with different challenges with respect to the generation of a conversion profile, as the output data from devices usually contain the data of several cycles, therefore the processing of the files needs to include a separation of the content according to multiple repetitions of reduction and oxidation in one study. According to the converter's routines, each cycle (including reduction and oxidation path) is converted to one jdx-file, allowing a clear separation

of the cycles while keeping the data in one container for a direct comparison. In case of non normalized CV measurements, the adjusted voltage and the measured current are plotted. Normalization (e.g. $\frac{I}{A}$) will be a feature provided by future software updates. Once the profile is created in the converter by the ELN admin, all files of the same type including the predefined identifier (Table 1), are processed automatically according the selected profile to a standardized BagIt bag including different jdx-files with xy-correlation of the single CV measurements. Altogether, we defined three profiles for the conversion of CV measurements, according to the three file types available from the aforementioned vendors. Other profiles could be configured easily in the future.

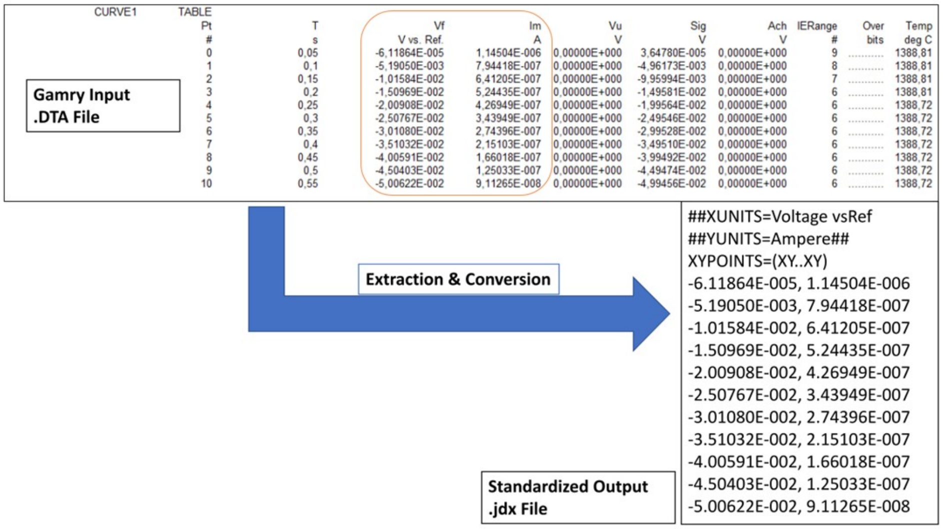

Figure S4: Visualization of conversion with ChemConverter from Gamry's DTA format to the open format JCAMP-DX.

3.1 Step 2b: Metadata mapping with ChemConverter in detail

A key step is the definition and implementation of a target metadata scheme, to which the extracted metadata - independent of the data source - are mapped to. The integration into an ELN as Chemotion offers the option to embed those target metadata schemes and thus providing the necessary mapping target.

To visualize, edit and add additional metadata to an analysis, a JAVA Script based GUI is used by the ELN. The GUI is highly flexible but bound to a specific ontology (e.g. CV, NMR, IR …). Users with special rights are able to build and edit the GUI with the help of a simple Web interface. Therefore buttons, fields and drop-down menus are defined and arranged. It’s also possible to define rules to hide specific fields or create ones with predefined units.

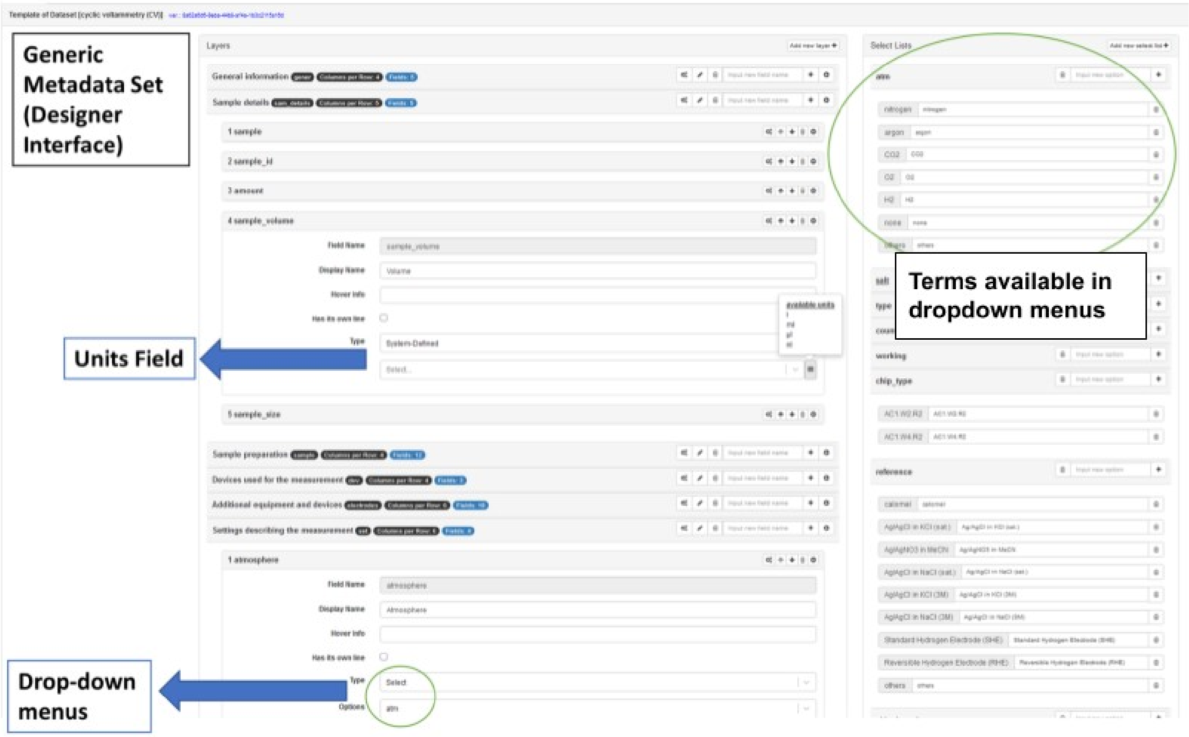

Figure S5: Screenshot of the *Generic Dataset* designer, a tool built to define metadata for each measurement type used in the ELN Chemotion/LabIMotion. Fields and elements created and

designed here are presented as input fields in the GUI of the ELN and can be autofilled during the file conversion process of ChemConverter. Different types of fields can be selected e.g. simple text, “system-defined” for physical units and “select” for drop-down menus for the user to choose from a preselected list later. The JSON implementation of the *Generic Dataset* can be gained from the supporting data deposition in Zenodo (https://doi.org/10.5281/zenodo.12827203).

We collected the required metadata for cyclic voltammetry measurements with representatives of the community as a basis for metadata mapping. The collected required information was defined as metadata form in the ELN to allow users to fill this information if directly available and it is provided as a target scheme for extracting and matching metadata that comes from a measurement. The design and representation of the metadata scheme was done with the GUI described in Figure S7. The metadata scheme consists of different sections, each bearing specific information on the used samples, the preparation of samples and the setting, the used devices and equipment and other conditions and parameters of the measurement. As the required metadata consist of usually data that can be gained from a file and needs to be completed with data given by the user (e.g. data describing the setting, including e.g. the type of electrodes, the conditions and other parameters of the measurement, can be added), a combination of extracted and matched metadata and users’ input seems to be suitable in most of the cases.

cyclic voltammetry (CV)

Layer Label	Field Label	Value	Unit	Name	Type	Source?	Source identifier	Source data
**General information**								
	Title			title	text			
	Data Type			data_type	text			
	Date	18.9.2023		date	text			
	Time	10:35:02		time	text			
	Contributor			contributor	text			
	Creator			creator	text			
	operator	DHM		operator	text			
	Comments			comments	text			
**Sample details**								
	label	DHM-15		label	text	Chemotion		DHM-15
	id	113176		id	text	Chemotion		113176
	Amount	1	mg	amount	system-defined-mass			
	Volume	0	l	sample_volume	system-defined-volumes			
	Size			size	text			
**Sample preparation**								
	Solvent	MeCN		solvent	select-solvents			
	Other solvent			solvent_other	text			
	Volume of solvent	2	ml	amount_sol	system-defined-volumes			
	Concentration of analyte	1	mmol_l	concentration	system-defined-molarity			
	Supporting electrolyte	TBAPF6		salt	select-salt			
	Other salt			salt_others	text			
	Amount supporting electrolyte	193	g	amount_salt	system-defined-mass			
	Concentration supporting electrolyte	100	mmol_l	concentration_salt	system-defined-molarity			
	Purge electrolyte solution	true		purge_sel	checkbox			
	Purge gas	nitrogen		purge	select-atm			
	Other purge gas			purge_other	text			
	Purge flow rate	0	ul_min	purge_flow	system-defined-flow_rate			
	Purge time	5	min	purge_time	system-defined-duration			
**Devices used for the measurement**								
	Brand	Gamry		brand	text			
	Serial No	IFC1010-30147		serial	text			
	Instrument	Potentiostat		instrument	text			
**Additional equipment and devices**								
	Used electrodes	traditional		type	select-type			
	ECSA method	geometric		ecsa_method	text			
	Polishing method	8 figure-eight		polishing_method	text			
	Polishing material	Al2O3		polishing_material	select-polishing_material			
	Other polishing materials			polishing_others	text			
	Particle size	0	mm	polishing_material_size	system-defined-length			
	Chip brand			chip	select-chip_brand			
	Chip type			chip_type	select-chip_type			
	Working electrode (WE)	glassy carbon		working	select-working			
	Other working electrode			working_others	text			
	WE-area [mm²]	0.785		working_area	text			
	Counter electrode (CE)	platinum		counter	select-counter			
	Other counter electrode			counter_others	text			
	CE-area [mm²]	2.010		counter_area	text			
	Reference electrode (RE)	others		reference	select-reference			
	Other reference electrode	Ag/AgNO3 (0.01 M AgN		reference_others	text			
	RE-area [mm²]			reference_area	text			
	Special coating	false		coating	checkbox			
	Coating material			coating_details	text			
**Settings describing the measurement**								
	Operation mode	without motion		mode	select-operation_mode			
	Atmosphere	nitrogen		atmosphere	select-atm			
	Other atmosphere			atmosphere_others	text			
	Temperature	0	C	temperature	system-defined-temperature			
	Separation	none		separation	select-separations			
	Conditions	dark		conditions	select-conditions			
	Wavelength			conditions_ill	text			
	Comments			comments	text			
**Measurement details**								
	Internal reference	ferrocene		reference_internal	select-reference_measure			
	Other internal reference			reference_internal_others	text			
	No of cycles	3		cycles	integer	Device		3
	Scan rate [V/s]	0.0999998		scan_rate	text	Device		0.0999998
	Step size [V]	0.005		step_size	text	Device		0.005
	Rotational rate [rpm]			rate	text			
	iR correction value [Ohm]			ir_two	text			
	iR correction included	false		ir	checkbox			
	Voltage start [V]	3,00000E-001		voltage_start	text	Device		3,00000E-001
	Voltage limit 1 [V]	-7,00000E-001		voltage_limit_one	text	Device		-7,00000E-001
	Voltage limit 2 [V]	3,00000E-001		voltage_limit_two	text	Device		3,00000E-001
	Voltage limit end [V]	3,00000E-001		voltage_limit_end	text	Device		3,00000E-001
**Plotting parameters**								
	Plotting convention	IUPAC		plotting_convention	select-plotting_conventions			
	xunits			xunits	text			
	yunits			yunits	text			
	xfactor			xfactor	text			
	yfactor			yfactor	text			
	resolution			resolution	text			
**Software used for data generation**								
	Software	Framework		software	text			
	Software version	7.8.6		software_version	text			

Figure S6: List of metadata that is available as a basic set of metadata as *.xlsx. The Metadata that can be extracted from the measurement file or completed manually in the ELN UI. The generated Excel file is also able to distinguish the source between device and user.

After the extraction and listing inside the Converter GUI, the desired metadata can be assigned to different key-values. This step allows the mapping of different sets of metadata from different original file types to just one standard metadata scheme.

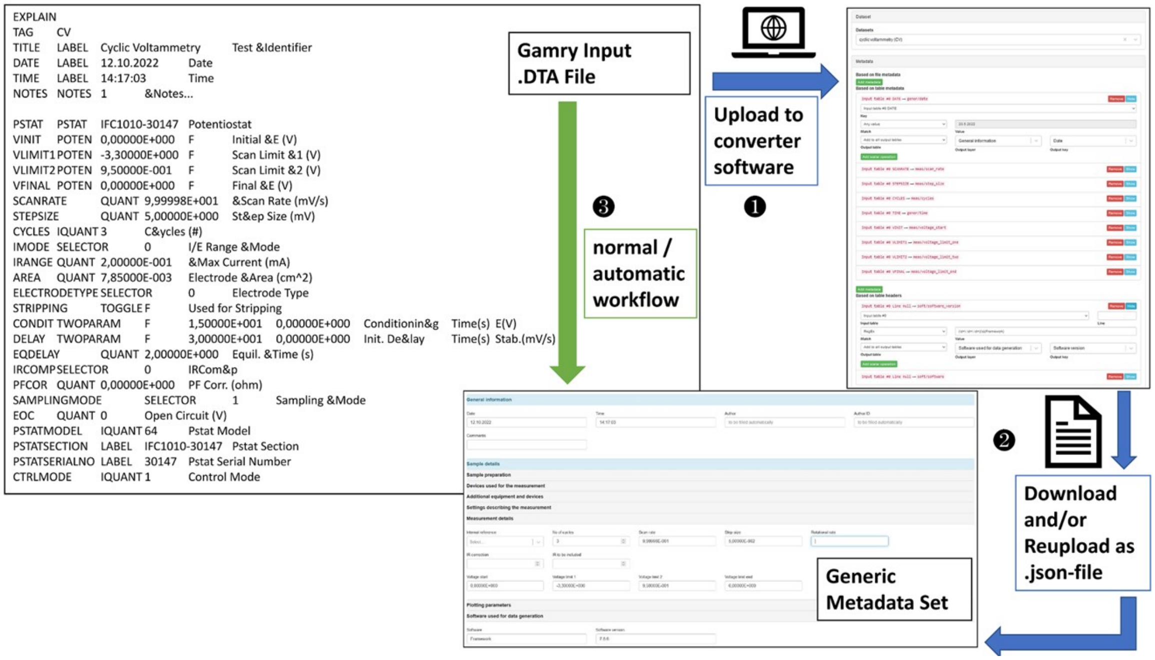

Figure S7: Depicted parts of the workflow combining the selection of metadata in the training input file, the mapping of the selected metadata to a metadata scheme and the visualization of the real results (= extracted metadata) after the upload of a real input file.

Example for downloaded table format

The results of the analyzed data from the data plot in ChemSpectra can be downloaded as a table format file.

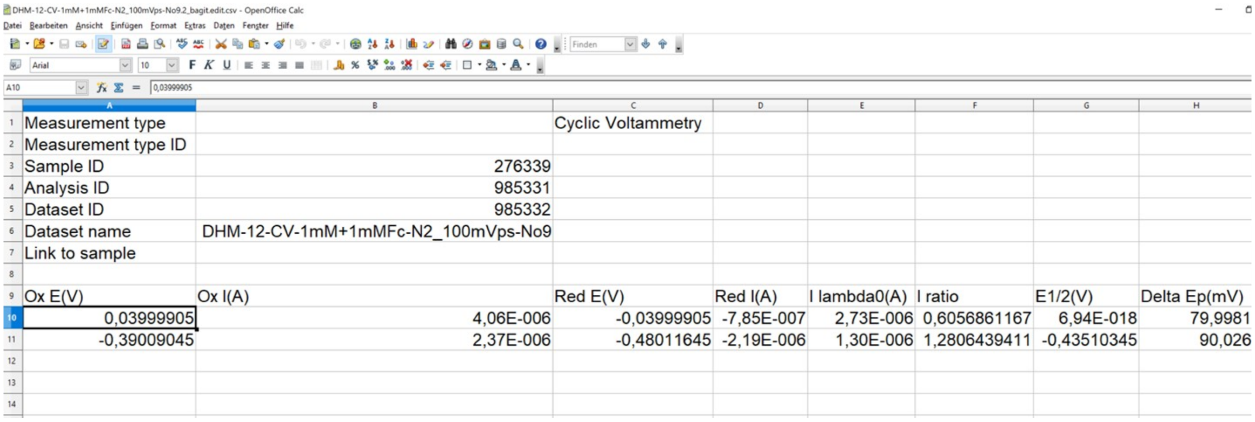

	A	B	C	D	E	F	G	H
1	Measurement type		Cyclic Voltammetry					
2	Measurement type ID							
3	Sample ID	276339						
4	Analysis ID	985331						
5	Dataset ID	985332						
6	Dataset name	DHM-12-CV-1mM+1mMFc-N2_100mVps-No9						
7	Link to sample							
8								
9	Ox E(V)	Ox I(A)	Red E(V)	Red I(A)	I lambda0(A)	I ratio	E1/2(V)	Delta Ep(mV)
10	0,03999905	4,06E-006	-0,03999905	-7,85E-007	2,73E-006	0,6056861167	6,94E-018	79,9981
11	-0,39009045	2,37E-006	-0,48011645	-2,19E-006	1,30E-006	1,2806439411	-0,43510345	90,026
12								
13								
14								

Figure S8: Screenshot of a csv output file from ChemSpectra. The tool produces one csv file for each curve/cycle of the experiment. It contains the peak values (potential and current) of the redox-

pairs defined by the user in the frontend as well as the calculated values (current ratio, half-wave potential and ΔE_p) derived from them. Each line in the table mirrors one redox-pair. In this example, the first line represents the attributes of the added ferrocene reference substance shifted to $E_{1/2}$ -> 0 V and the second line represents the features of the measured complex sample.

4 BagIt Implementation

To achieve a standardized and structured output container, BagIt is used. BagIt is a hierarchical file system conventions created by the California Digital Library and the Library of Congress[5]. BagIt is about reliable transfer of content in a package, which are called “bags”. It is also possible to serialize a bag as a zip-file if a single file for uploading is needed. According to the memo from the IETF the bag consists of required and optional elements. Required are the “bagit.txt” file and a data / payload sub-directory. The bagit.txt contains a line with the BagIt version (in our case 1.0) and a line with the used encoding (UTF-8) of the tags. Also at least one manifest tag file is needed named after the checksum algorithm and containing a checksum and path for each file in the payload directory. We decided to use sha256 and sha512 simultaneously. Two different algorithms are used for the purpose of double checking [2]. The “bagit.txt” must contain the used BagIt version and encoding which is 1.0 and UTF-8. Each recorded curve or cycle of the CV data is stored as one file in JCAMP-DX format after conversion inside the “data” folder while the converted metadata is stored as a .json-file inside “metadata/converter.json”. To avoid problems with up- and downloading multiple files, the BagIt container is serialized as .zip-file.

5 Special features for Cyclic Voltammetry in ChemSpectra

ChemSpectra further allows the calculation and assignment of some standardized key values to describe and compare different curves of CV experiments when using different references or

equipment (e.g. the voltage and current absolute values could be quite different if using other electrodes). The most common and already implemented values are

- the correction value of the capacitive background current aka $I_{\square 0}$ (or sometimes also i_{sp0}). This value is currently determined graphically by the user.
- The current (I in Ampere) ratio i_{pa}/i_{pc} of a given redox-pair calculated by the approximation formula of NICHOLSON, Rl S. 1966. Adopted to ChemSpectra's notation the formula is calculated as: $I_{ratio} = \frac{|I_{p,min}|}{|I_{p,max}|} + \frac{(0.485) \times |I_{\lambda o}|}{|I_{p,max}|} + 0.086$ whereby I_p are the current values of the anodic and cathodic peaks.
- The peak separation between the anodic and cathodic peak $\Delta E_p = |E_{p,max} - E_{p,min}|$ whereby E is the voltage values of the peaks.

6 ChemSpectra Workflow

The interaction between users and their data files within the ChemSpectra and ELN ecosystem is illustrated in Figure S9 as a flow diagram. As an integrated application, ChemSpectra manages data transfer between the ELN, its own front-end and back-end systems, and the user interface. This process involves multiple iterations of data exchange and ensures seamless integration, traceability and functionality.

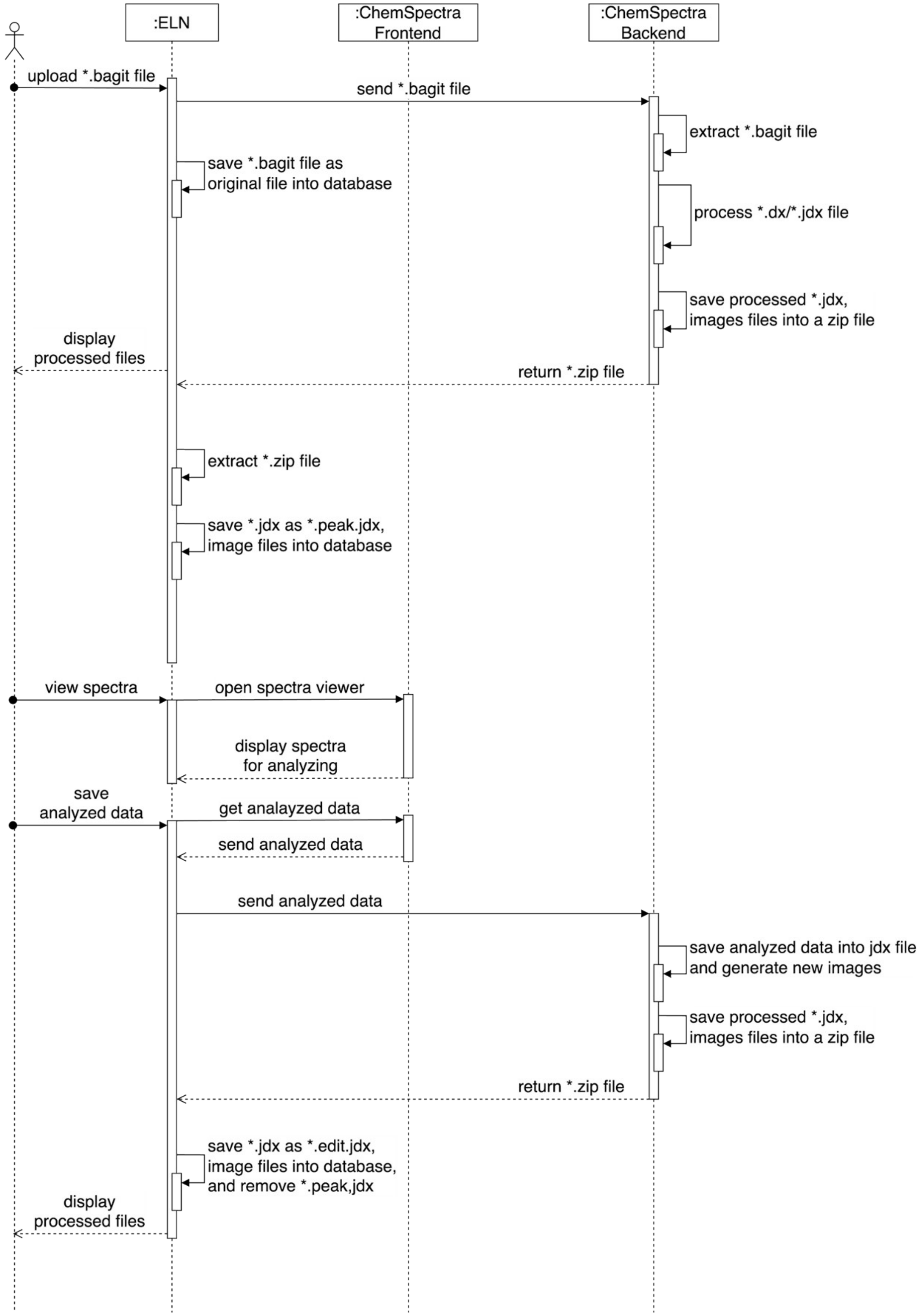

Figure S9: Complete workflow diagram of *ChemSpectra* and the interaction of its front- and backend with the ELN and the user.

7 Inline Notation

We also provide a draft for an inline notation to report redox potentials obtained by CV measurements, similar to the so-called NMR textual analysis, initially defined by two IUPAC Recommendations[6,7]. For NMR, this inline notation is generated automatically by *ChemSpectra* after analysis of NMR data in *ChemSpectra*. The same will apply to CV in an upcoming release of Chemotion ELN. After several reviews within the authors of this document we agreed on the following inline notation, taking $Cu(TMGqu)_2PF_6$ as an example:

CV (1 mM in MeCN vs. Ref (Fc^+/Fc) = 0.38 V, ν = 0.1 V/s, to neg.):

$E_{1/2}$ ($[Cu(TMGqu)_2]^{2+}/[Cu(TMGqu)_2]^+$, ΔE_p) = −0.04 V (80 mV).

Similar to the NMR text, the inline notation starts with the abbreviation of the method used. This is followed in parentheses by information on the concentration of the analyte and the solvent applied. In addition, the redox couple of the reference substance is mentioned together with the explicit mention of the value of the redox potential of the reference substance used for calibration i.e. to which reference the redox potentials are *reported*. The signal of the redox couple of the reference substance is regularly set to 0 V, which is common practice among some of the authors of this work, but it can be set to any other value, but this should be reported redox potentials of organic molecules are regularly reported vs. SCE, while the actual measurement was referenced with ferrocene as internal standard. Following Pavlishchuk and Addison the redox potential of the Fc^+/Fc couple is +0.38 V vs. SCE[8] and taken as reference for the example provided above. The reference or quasi reference electrode (QRE)[9],[10] used in the measurement cell is considered to be part of the metadata (see above, Generic Dataset Template), if the measurement is internally referenced using ferrocene or any other internal standard. Moreover, the inline notation provides

information on the scan rate ν (lowercase Nu, following IUPAC Recommendations[9] and IUPAC Orange Book) as well as the direction the measurement was started (to neg./ to pos.). The latter information was considered to be important as reactive (radical) intermediates formed within the cell might induce additional redox events in CV spectra[11] (e.g. dehalogenation of arylhalides). Following the parentheses, the half-wave potential $E_{1/2}$ for each redox event is provided. The redox couple is given in parentheses according to IUPAC Green Book 2007[12], followed by ΔE_p, separated by a comma. For irreversible redox events, where the potential may be determined based on half-peak potentials $E_{p/2}$[10], ΔE_p and the corresponding parentheses and the value are omitted. For redox quiet counter ions (if present), these counter ions may be omitted. The half-wave potential(s) $E_{1/2}$ in V are subsequently reported after the equal sign together with the ΔE_p in mV in parentheses. Each reported redox couple with its redox potential reported is separated with a comma plus single space and each notation ends with a full stop.

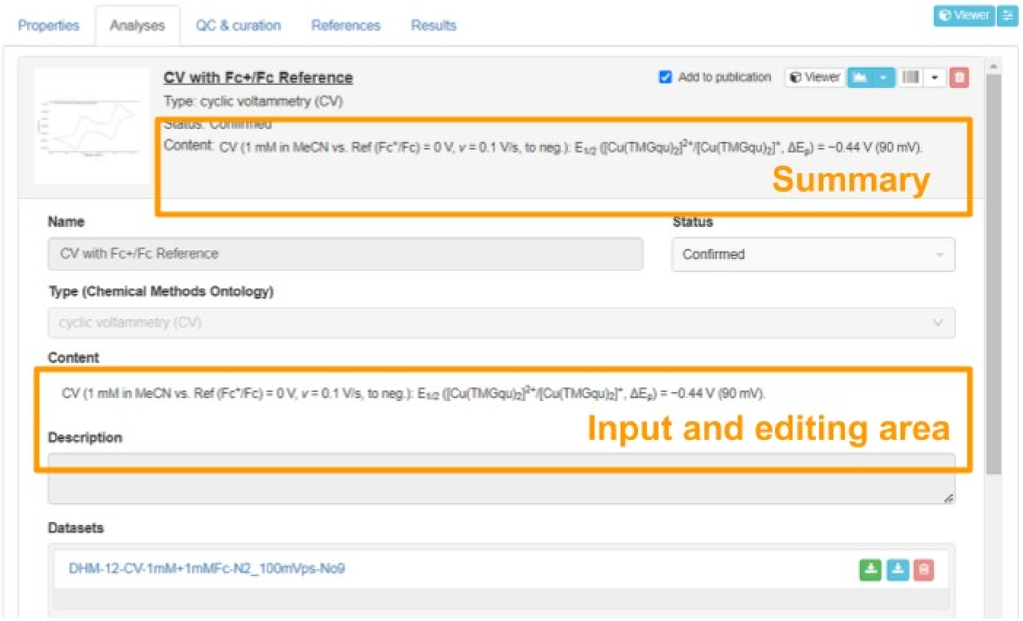

Figure S10: Implementation of the inline notation in the analysis section of the Chemotion ELN and Chemotion Repository.

P
A

8 Representation of data in different repositories

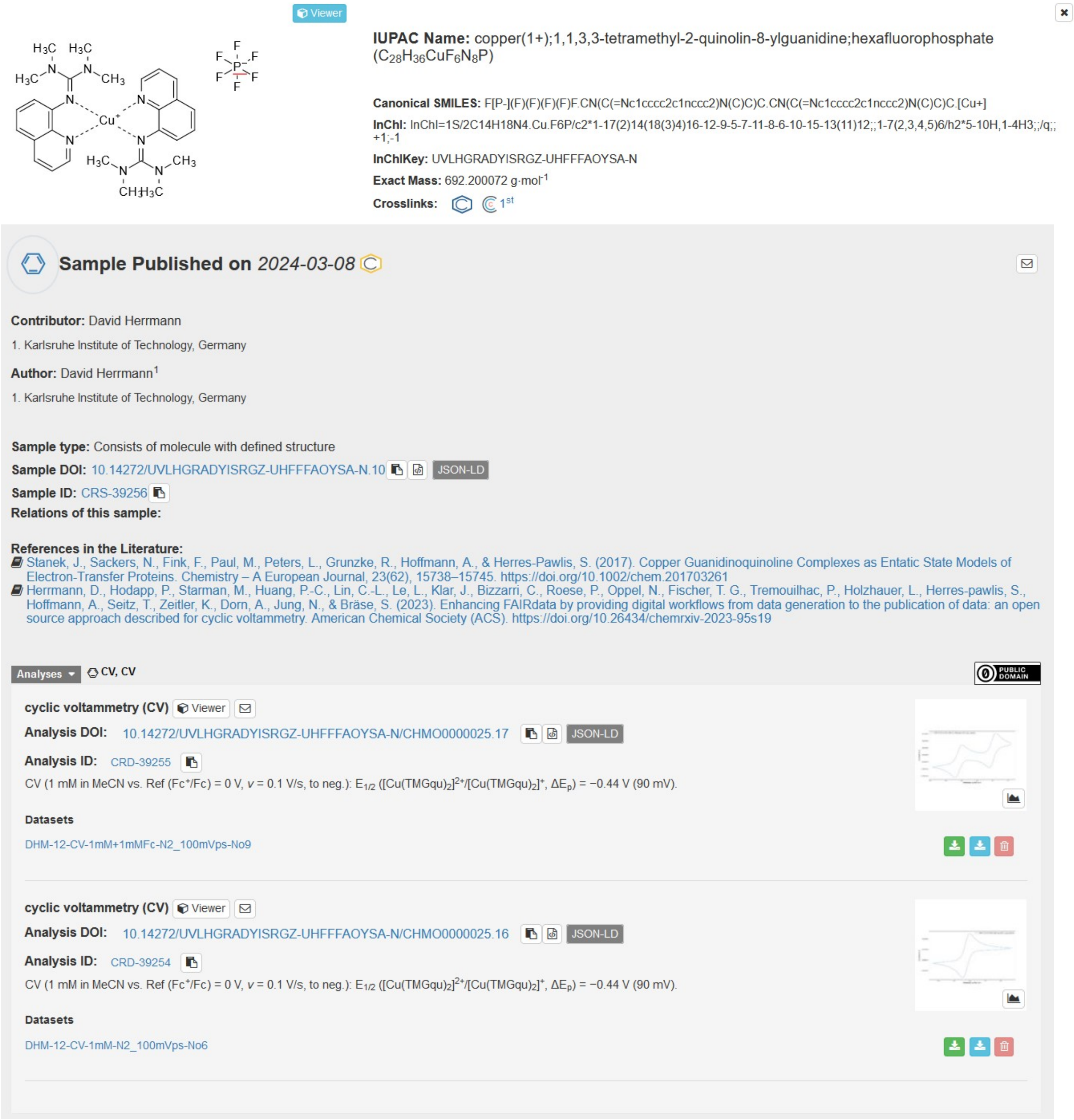

Figure S11: Representation of the same dataset in three different repositories: CV dataset in the Chemotion Repository (https://dx.doi.org/10.14272/UVLHGRADYISRGZ-UHFFFAOYSA-N.10) .

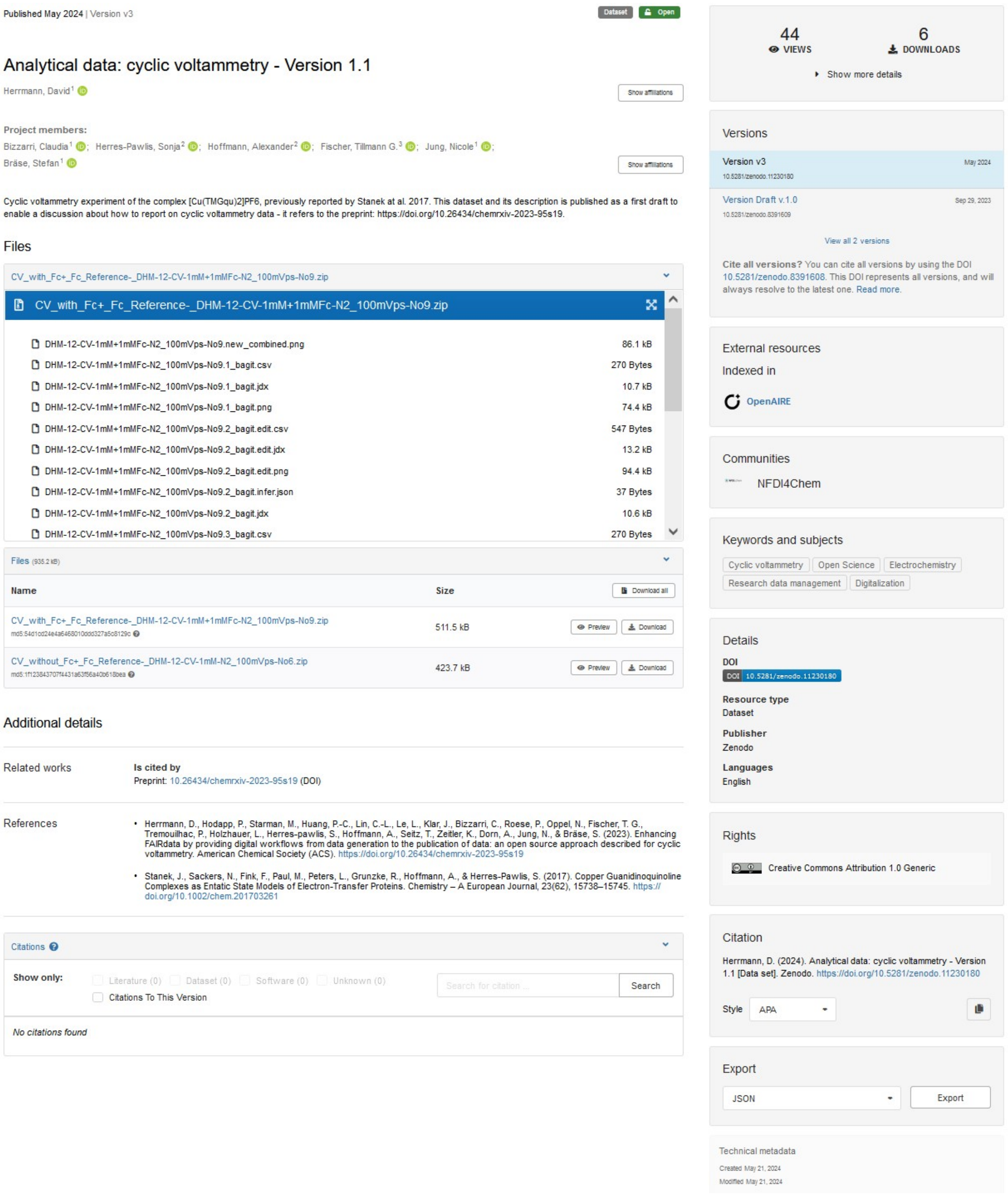

Figure S12: Representation of the same dataset in three different repositories: CV dataset in Zenodo (https://doi.org/10.5281/zenodo.11230180).

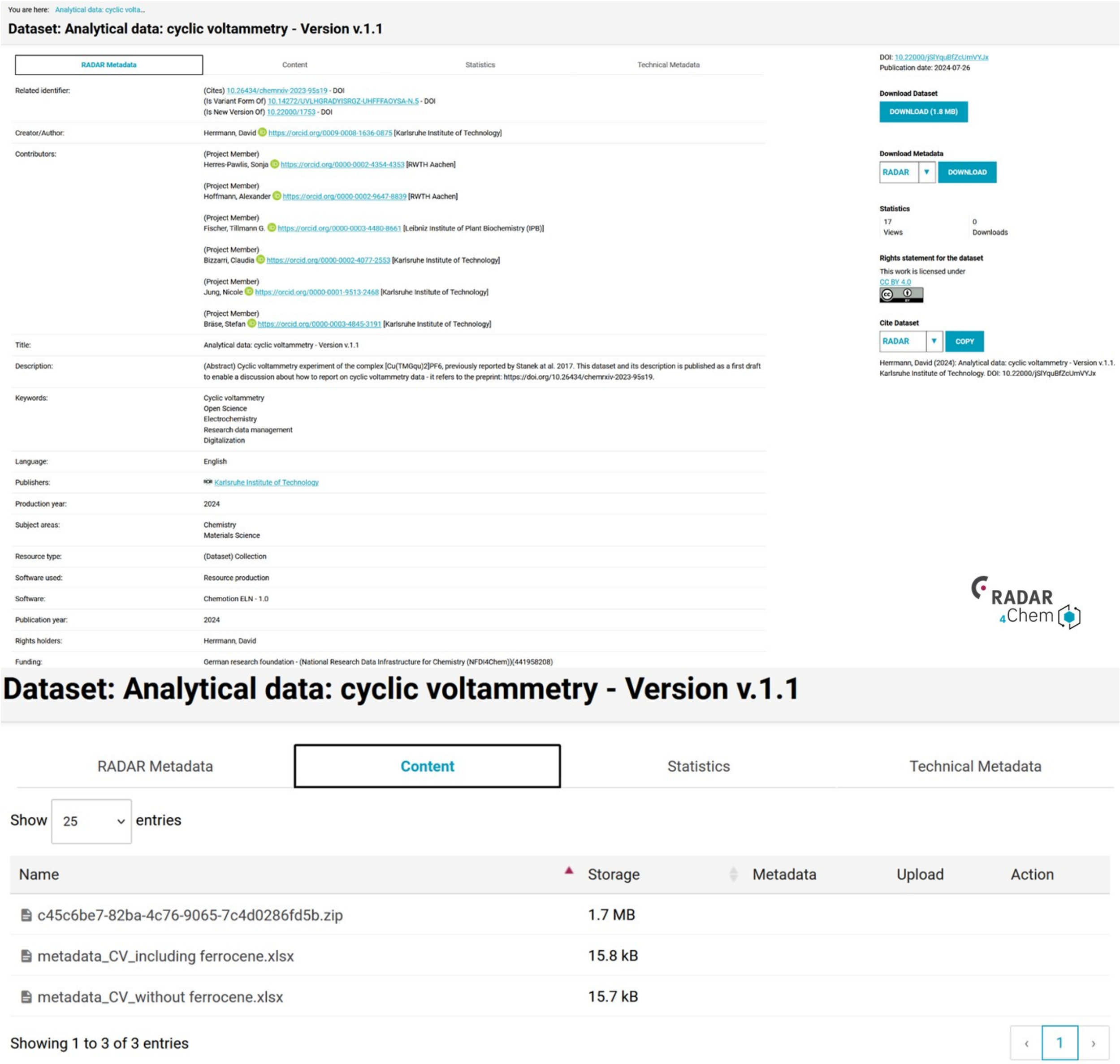

Figure S13: Representation of the same dataset in three different repositories: CV dataset in RADAR4Chem (https://dx.doi.org/10.22000/jSlYquBfZcUmVYJx). The logo was arranged.

Table 2. List of the DOI-Links for the different Chemotion repository entries published by the diverse research facilities for the same compound. Following the links for the sample leads to the same molecule page in the Chemotion repository but on the right spot, while following the link for

an analysis shows an extra page only showing the wanted analysis together with the sample's structure (see Figure S14).

Side	DOI for sample	DOI for analysis
Institute of Biological and Chemical Systems – Functional Molecular Systems (IBCS-FMS), KIT, Germany	https://dx.doi.org/10.14272/UVLHGRADYISRGZ-UHFFFAOYSA-N.10	https://dx.doi.org/10.14272/UVLHGRADYISRGZ-UHFFFAOYSA-N/CHMO0000025.17
Institute for Applied Materials – Electrochemical Technologies (IAM-ET), KIT, Germany	https://dx.doi.org/10.14272/UVLHGRADYISRGZ-UHFFFAOYSA-N.8	https://dx.doi.org/10.14272/UVLHGRADYISRGZ-UHFFFAOYSA-N/CHMO0000025.14
Institute of Organic Chemistry (IOC), KIT, Germany	https://dx.doi.org/10.14272/UVLHGRADYISRGZ-UHFFFAOYSA-N.7	https://dx.doi.org/10.14272/UVLHGRADYISRGZ-UHFFFAOYSA-N/CHMO0000025.11
University - Institute of Organic Chemistry & Institute of Plant Biochemistry - Bioinformatics & Scientific Data, Leipzig, Germany	https://dx.doi.org/10.14272/UVLHGRADYISRGZ-UHFFFAOYSA-N.11	https://dx.doi.org/10.14272/UVLHGRADYISRGZ-UHFFFAOYSA-N/CHMO0000025.18 (*)
Chair of Bioinorganic Chemistry and Institute of Inorganic Chemistry (IAC), RWTH Aachen University, Germany	https://dx.doi.org/10.14272/UVLHGRADYISRGZ-UHFFFAOYSA-N.14	https://dx.doi.org/10.14272/UVLHGRADYISRGZ-UHFFFAOYSA-N/CHMO0000025.21 (**)

(*) The PSSESSION format of the PalmSens device used by the group in Leipzig University is able to hold data of more than one measurement, so they are able to submit one analysis datafile containing a measurement with Ferrocene added and a measurement without Ferrocene. (**) The group from Aaachen only submitted the measurement containing the compound only.

The submission of the alternative compound could also be found in the Chemotion repository using this DOI: https://dx.doi.org/10.14272/PHXDKLQHLVWUNE-UHFFFAOYSA-N.3

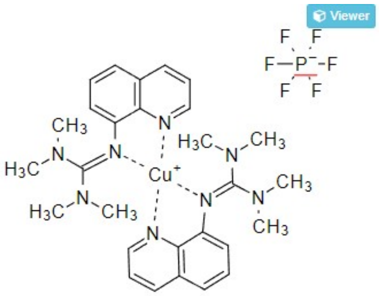

This information is based on the molecular structure shown on the left side. For a decoupled sample, please refer to its individual details.

Formula: $C_{28}H_{36}CuF_6N_8P$

Canonical SMILES: F[P-](F)(F)(F)(F)F.CN(C(=Nc1cccc2c1nccc2)N(C)C)C.CN(C(=Nc1cccc2c1nccc2)N(C)C)C.[Cu+]

InChI: InChI=1S/2C14H18N4.Cu.F6P/c2*1-17(2)14(18(3)4)16-12-9-5-7-11-8-6-10-15-13(11)12;;1-7(2,3,4,5)6/h2*5-10H,1-4H3;;/q;;+1;-1

InChIKey: UVLHGRADYISRGZ-UHFFFAOYSA-N

Exact Mass:

Crosslinks:

Published on *2024-03-08*

PUBLIC DOMAIN

cyclic voltammetry (CV)

Author: David Herrmann[1]

1. Karlsruhe Institute of Technology, Germany

Analysis DOI: 10.14272/UVLHGRADYISRGZ-UHFFFAOYSA-N/CHMO0000025.17 JSON-LD

Analysis ID: CRD-39255

Content: CV (1 mM in MeCN vs. Ref (Fc+/Fc) = 0 V, v = 0.1 V/s, to neg.): E1/2 ([Cu(TMGqu)2]2+/[Cu(TMGqu)2]+, ΔEp) = −0.44 V (90 mV).

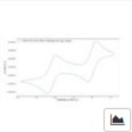

Datasets

DHM-12-CV-1mM+1mMFc-N2_100mVps-No9

Published on *2024-03-08*

PUBLIC DOMAIN

cyclic voltammetry (CV)

Author: Niklas Oppel[1]

1. Institute for Applied Materials – Electrochemical Technologies (IAM-ET), Karlsruhe Institute of Technology, Germany

Analysis DOI: 10.14272/UVLHGRADYISRGZ-UHFFFAOYSA-N/CHMO0000025.14 JSON-LD

Analysis ID: CRD-39219

Content: CV (1 mM in MeCN vs. Ref (Fc+/Fc) = 0 V, v = 0.1 V/s, to pos.): E1/2 ([Cu(TMGqu)2]2+/[Cu(TMGqu)2]+, ΔEp) = -0.43 V (80 mV)

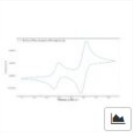

Datasets

CV_CuTMGqu_04_positiv_mitFC

P
A

Published on *2024-03-08*

cyclic voltammetry (CV)

Author: Claudia Bizzarri[1]

1. Karlsruhe Institute of Technology, Deutschland

Analysis DOI: 10.14272/UVLHGRADYISRGZ-UHFFFAOYSA-N/CHMO0000025.11 JSON-LD

Analysis ID: CRD-39102

Content: CV (1 mM in MeCN vs. Ref (Fc+/Fc) = 0 V, v = 0.1 V/s, to neg.): E1/2 ([Cu(TMGqu)2]2+/[Cu(TMGqu)2]+, ΔEp) = −0.43 V (78 mV).

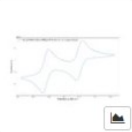

Datasets

CBz_20230907_ACN_Cu(TMGqu)2PF6_100 mVs-1_Fc

Published on *2024-03-12*

cyclic voltammetry (CV)

Authors: Alrik Dorn[1] - Tillmann G. Fischer[2] - Kirsten Zeitler[1]

1. Institute of Organic Chemistry, Leipzig University, Germany

2. Bioinformatics & Scientific Data, Leibniz Institute of Plant Biochemistry, Germany

Analysis DOI: 10.14272/UVLHGRADYISRGZ-UHFFFAOYSA-N/CHMO0000025.18 JSON-LD

Analysis ID: CRD-39581

Content: CV (1 mM in MeCN vs. Ref (Fc+/Fc) = 0.38 V, v = 0.1 V/s, to pos.): E1/2 ([Cu(TMGqu)2]2+/[Cu(TMGqu)2]+, ΔEp) = −0.04 V (80 mV).

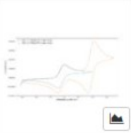

Datasets

ADO-1_CuTMGqu2PF6

Published on *2024-07-29*

cyclic voltammetry (CV)

Authors: Alexander Hoffmann[1] - Sonja Herres-Pawlis[2]

1. Institute of Inorganic Chemistry, RWTH Aachen University, Germany

2. Fachgruppe Chemie, Rheinisch Westfälische Technische Hochschule Aachen, Germany

Analysis DOI: 10.14272/UVLHGRADYISRGZ-UHFFFAOYSA-N/CHMO0000025.21 JSON-LD

Analysis ID: CRD-43416

Content: CV (1 mM in MeCN vs. Ref (Fc+/Fc) = 0 V, v = 0.1 V/s, to neg.): E1/2 ([Cu(TMGqu)2]2+/[Cu(TMGqu)2]+, ΔEp) = -0.46 V (73 mV).

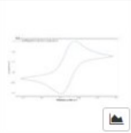

Datasets

CV of [Cu(TMGqu)2] by 100mV/s

P
A

Figure S14: Representation of an analysis in Chemotion Repository when following the associated DOI-Link. The upper part, which shows the structural formula of the molecule, is identical for all analyses and is therefore only shown once.

A comparison of Figures S11 to S14 shows that the Chemotion repository has a clear advantage in the representation of the relationship between the analysis data set and the assigned sample due to its architecture.

9 References

1. Tremouilhac, P. *et al.* Chemotion ELN: an Open Source electronic lab notebook for chemists in academia. *J. Cheminform.* **9**, 54 (2017).
2. Potthoff, J. *et al.* Procedures for systematic capture and management of analytical data in academia. *Anal Chim Acta X* **1**, 100007 (2019).
3. Huang, Y.-C., Tremouilhac, P., Nguyen, A., Jung, N. & Bräse, S. ChemSpectra: a web-based spectra editor for analytical data. *J. Cheminform.* **13**, 8 (2021).
4. Tremouilhac, P. *et al.* The Repository Chemotion: Infrastructure for Sustainable Research in Chemistry*. *Angew. Chem. Int. Ed Engl.* **59**, 22771–22778 (2020).
5. Kunze, J., Littman, J., Madden, E., Scancella, J. & Adams, C. *The BagIt File Packaging Format (V1.0)*. https://www.rfc-editor.org/rfc/rfc8493 (2018) doi:10.17487/rfc8493.
6. Recommendations for the Presentation of NMR Data for Publication in Chemical Journals. *J. Macromol. Sci. Part A Pure Appl. Chem.* **29**, 625–628 (1972).
7. Presentation of NMR Data for Publication in Chemical Journals - B. Conventions Relating to Spectra from Nuclei Other Than Protons. *De Gruyter*

https://www.degruyter.com/database/IUPAC/entry/iupac.45.0031/html.

8. Pavlishchuk, V. V. & Addison, A. W. Conversion constants for redox potentials measured versus different reference electrodes in acetonitrile solutions at 25°C. *Inorganica Chim. Acta* **298**, 97–102 (2000).

9. Pingarrón, J. M. *et al.* Terminology of electrochemical methods of analysis (IUPAC Recommendations 2019). *J. Macromol. Sci. Part A Pure Appl. Chem.* **92**, 641–694 (2020).

10. *Compendium of Terminology in Analytical Chemistry*. (The Royal Society of Chemistry, 2023). doi:10.1039/9781788012881.

11. Sandford, C. *et al.* A synthetic chemist's guide to electroanalytical tools for studying reaction mechanisms. *Chem. Sci.* **10**, 6404–6422 (2019).

12. International Union of Pure and Applied Chemistry. Physical and Biophysical Chemistry Division. *Quantities, Units and Symbols in Physical Chemistry*. (Royal Society of Chemistry, 2007). doi:10.1039/9781847557889.

P
A
